# TINAGL1 and B3GALNT1 are potential therapy target genes to suppress metastasis in non-small cell lung cancer

**DOI:** 10.1186/1471-2164-15-S9-S2

**Published:** 2014-12-08

**Authors:** Hideaki Umeyama, Mitsuo Iwadate, Y-h Taguchi

**Affiliations:** 1 Department of Biological Science, Chuo University, 1-13-27 Kasuga, Bunkyo-ku, Tokyo 112-8551, Japan; 2 Department of Physics, Chuo University, 1-13-27 Kasuga, Bunkyo-ku, Tokyo 112-8551, Japan

**Keywords:** Gene expression, promoter methylation, integrated analysis, unsupervised feature selection, non-small cell lung cancer, metastasis, principal component analysis, *in silico *drug discovery, protein structure prediction

## Abstract

**Background:**

Non-small cell lung cancer (NSCLC) remains lethal despite the development of numerous drug therapy technologies. About 85% to 90% of lung cancers are NSCLC and the 5-year survival rate is at best still below 50%. Thus, it is important to find drugable target genes for NSCLC to develop an effective therapy for NSCLC.

**Results:**

Integrated analysis of publically available gene expression and promoter methylation patterns of two highly aggressive NSCLC cell lines generated by *in vivo *selection was performed. We selected eleven critical genes that may mediate metastasis using recently proposed principal component analysis based unsupervised feature extraction. The eleven selected genes were significantly related to cancer diagnosis. The tertiary protein structure of the selected genes was inferred by Full Automatic Modeling System, a profile-based protein structure inference software, to determine protein functions and to specify genes that could be potential drug targets.

**Conclusions:**

We identified eleven potentially critical genes that may mediate NSCLC metastasis using bioinformatic analysis of publically available data sets. These genes are potential target genes for the therapy of NSCLC. Among the eleven genes, TINAGL1 and B3GALNT1 are possible candidates for drug compounds that inhibit their gene expression.

## Background

Currently, there is no effective therapy for non-small cell lung cancer (NSCLC), thus NSCLC remains lethal [1]. The 5-year survival rate is at best still below 50%. In addition, NSCLC consists of several subtypes that require distinct therapies. Thus, from both a diagnosis and therapy point of view, the identification of genes critical to NSCLC is urgent. Few studies have identified NSCLC critical genes. Fawdar et al [2] recently found that mutations in *FGFR4, MAO3K *and *PAK5 *have critical roles in lung cancer progression. Li et al [3] also recently identified *EML4-ALK *fusion gene and *EGFR *and *KRAS *gene mutations were associated with NSCLC. Takeuchi et al [4] also reported that *RET, ROS1 *and *ALK *gene fusions were observed in lung cancer. However, it is likely that other critical gene candidates for NSCLC exist.

In this study, we attempted to identify new critical candidate genes important for NSCLC using recently proposed principal component (PCA) based unsupervised feature extraction (FE) mediated integrated analysis [5-8] of publically available promoter methylation and gene expression patterns of two NSCLC cell lines with and without enhanced metastasis ability.

In contrast to the standard usage of PCA, PCA based unsupervised FE does not embed samples but features (that is, probes in this study) into a low dimensional space. Then, features identified as outliers are extracted (for details, see method). Empirically this methodology was successful and identified biologically significant features [5-8] even when other conventional methods tested in the current study failed.

Most of the genes identified in the present study by this methodology were also previously reported as significant cancer-related genes. To understand the functionality of the selected genes, we predicted the tertiary structures of selected genes by Full Automatic Modeling System (FAMS) [9] and phyre2 [10] profile-based protein structure prediction software. This system also allowed the identification of drug target candidate genes.

## Results

### The first principal components show no significant difference between samples

**Figure 1:**
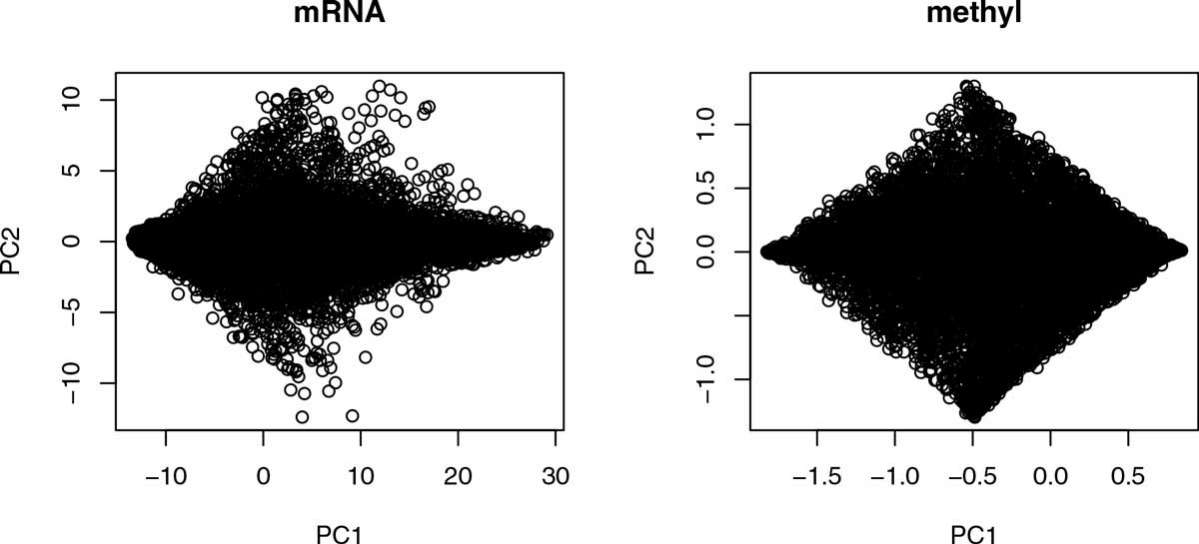
**Two-dimensional embeddings of probes using PCA**. Two-dimensional embeddings of probes (left: gene expression, right: promoter methylation) spanned by the first (horizontal axes) and the second (vertical) axes.

**Figure 2:**
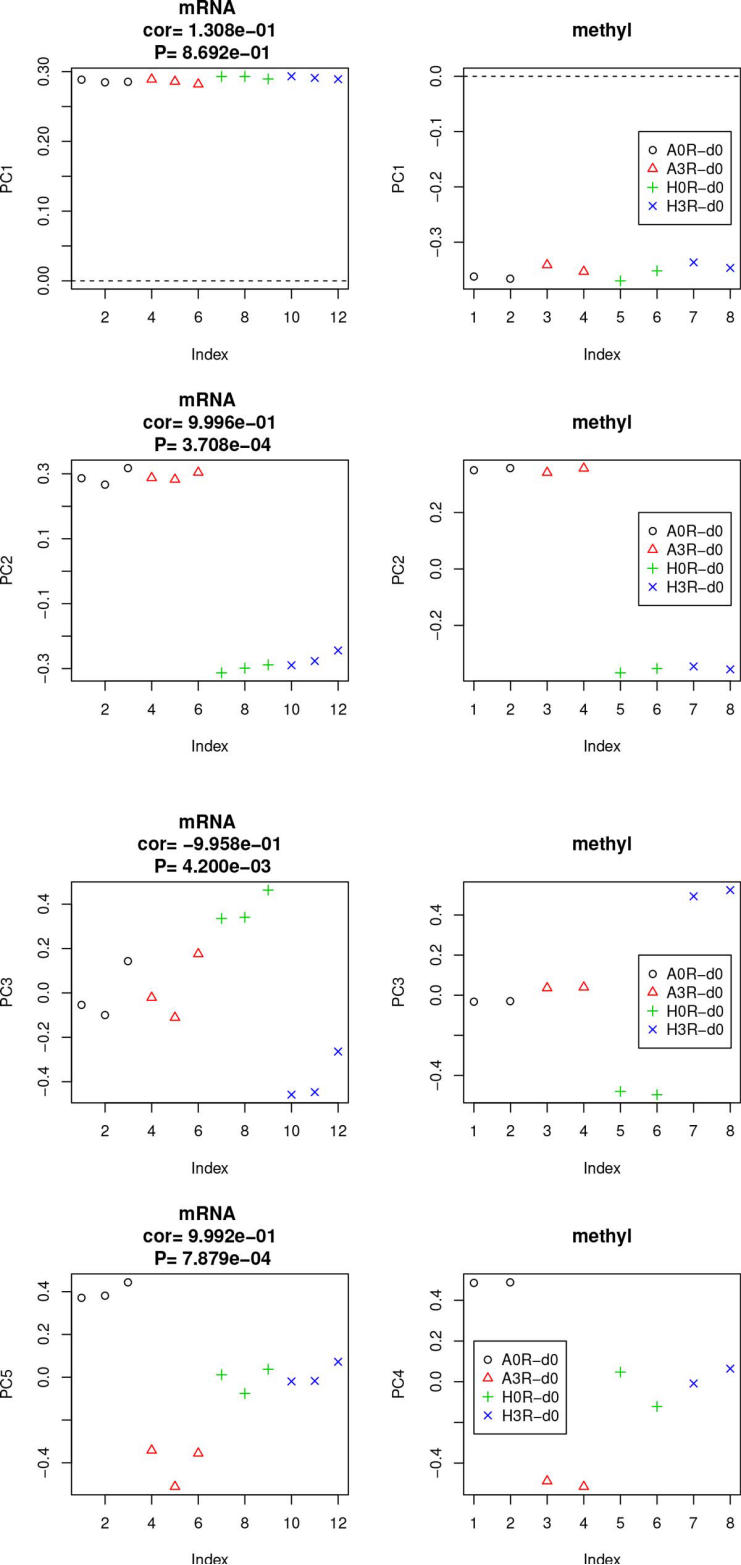
**Contributions of samples to PCs**. Contributions of samples (black open circles: A549 without metastasis, red triangles: A549 with metastasis, green crosses: HTB56 without metastasis, blue crosses: HTB56 with metastasis) to PCs. Left column: gene expression, right column: promoter methylation. "cor" indicates Pearson correlation coefficients of PCs between gene expression and promoter methylation averaged within each of four categories and "P" is attributed to "cor".

Since conventional methodology was not useful for the identification of differences between samples, we decided to employ PCA based unsupervised FE to extract biologically relevant genes (probes) when cell lines with and without metastasis were compared. Since it was not our main purpose to emphasize novelty and superiority of the present method compared with the conventional methods, but to identify critical genes for NSCLC metastasis, how the conventional methods failed to identify critical genes in NSCLC metastasis will be discussed in Discussion sections below (the section "Superiority and novelty of the proposed method"). Figure 1 shows two-dimensional embeddings of probes using PCA for gene expression and promoter methylation. To apply PCA based unsupervised FE, first we identified PCs to be used for FE. To determine what each principal component (PC) represented, the contributions of samples to the first PC (PC1) are shown (Figure 2). As previously observed [6,8], the first PC did not identify distinct features among the samples, although they had major contributions (97% for gene expression and 87% for promoter methylation). Contributions of samples to PC1 are almost constantly independent of samples for gene expression and promoter methylation. Thus, we concluded that PC1 did not exhibit any significant differences among samples. It should be noted that this does not always mean that PC1s are biologically meaningless, but rather that most gene expression and promoter methylation is sample independent; thus, the cell lines are very similar to each other independent of their ability for metastasis. This is not surprising, as they are similar NSCLC cell lines; therefore, significantly different outcomes caused by sample dependence and/or metastasis presence would be unusual.

### The second PCs demonstrates distinction between cell lines

**Figure 3:**
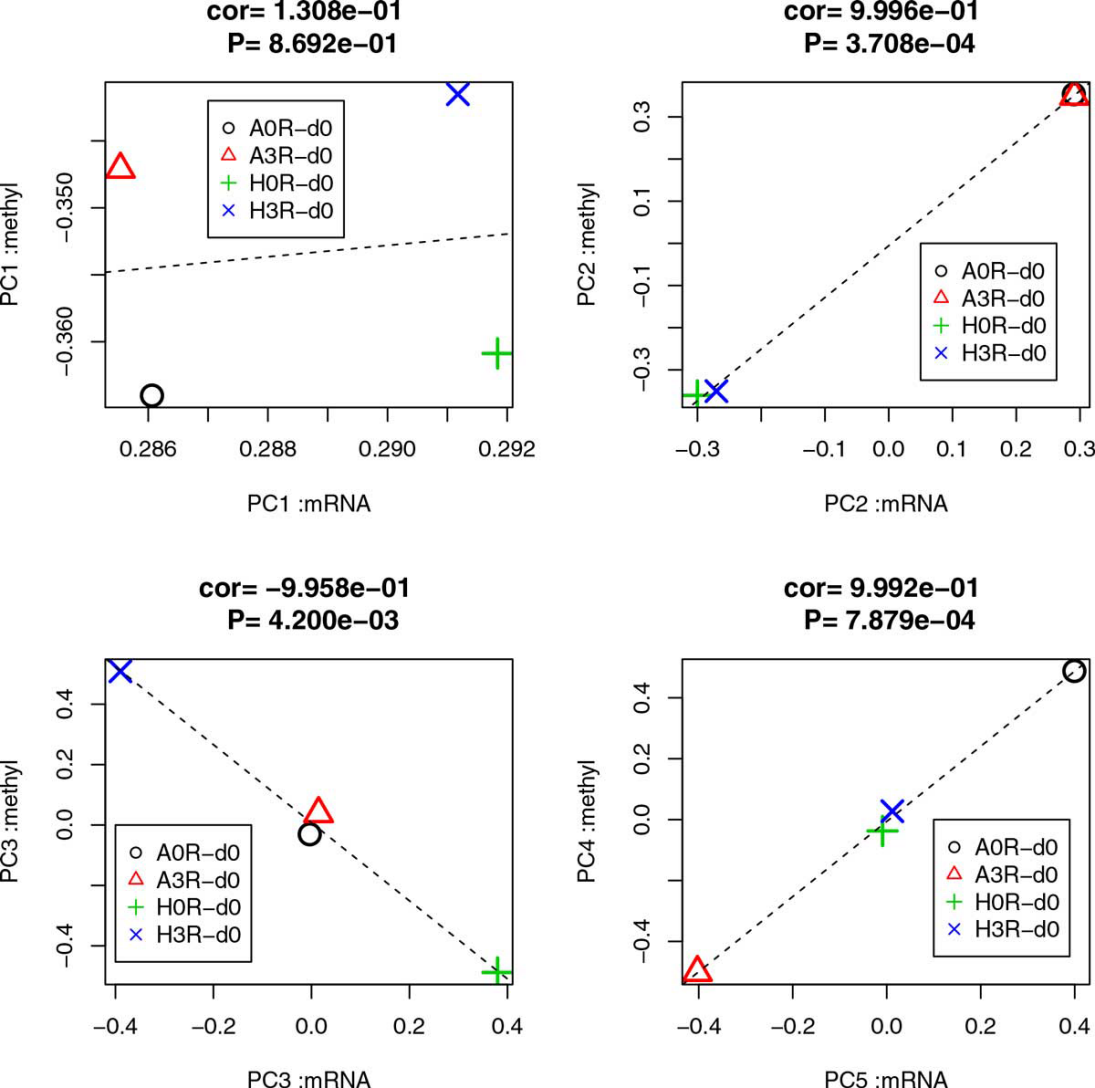
**Scatter plots of contributions of samples to PCs between gene expression and promoter methylation**. Scatter plots of PCs averaged within each of four categories. Contributions of samples (black open circles: A549 without metastasis, red triangles: A549 with metastasis, green crosses: HTB56 without metastasis, blue crosses: HTB56 with metastasis) to PCs. Left column: gene expression, right column: promoter methylation. "cor" indicates Pearson correlation coefficients of PCs between gene expression and promoter methylation averaged within each of four categories and "P" is attributed to "cor".

Because the first PCs did not distinguish between samples, we next considered second PCs (PC2s). As can be seen by two-dimensional embeddings of probes (Figure 1), the second PCs had a relatively smaller contribution. However, this does not always mean that PC2s are biologically meaningless, since PC2 having common values for all samples simply means that all samples behave similarly which does not always contradict with biological significance if all samples are equally biologically significant in the very similar fashions. The second PC of gene expression has only a 1.7% contribution, while for promoter methylation it is 9.6%. These values for contributions, especially for gene expression, can usually be ignored. However, in this case, since the samples were similar NSCLC cell lines, differences between samples were expected to be small. Thus, PCs with small contributions may represent biologically critical differences between samples, as shown in Figure 2, where the contributions of samples to PC2s are demonstrated. PC2s did not distinguish between samples with and without metastasis ability, but did distinguish between A549 and HTB56 cell lines. Because we are interested in metastasis-causing genes in HSCLC, what PC2 expresses is out of the scope of the present study. However, it is useful to identify genes associated with PC2 to determine which genes are different between the two cell lines, A549 and HTB56. PC2s showed good correlation between gene expression and promoter methylation (Figure 3). Thus, integrated analysis using PCA based unsupervised FE is applicable. Selected genes are shown in Table 1. Figure S1 (Additional file 1) shows the gene expression and promoter methylation of selected genes. If analysis is successful, genes selected based on mRNA expression and those based on promoter methylation should overlap significantly. Indeed, *P*-values attributed to selected genes common between gene expression and promoter methylation were 4.1×10^-9 ^and 5.1×10^-12^, respectively. Thus, integrated analysis using PCA based unsupervised FE was successful. In contrast to expectations, the selected genes were frequently and significantly related to cancers by the Gendoo server [11] (Table 1), which was used because it attributed *P*-values to each association between genes and disease. Thus, the reliability of associations can be more easily identified. The successful identification of associations between genes and disease by the Gendoo server suggests that HTB56 and A549 cell lines are potentially distinct to each other and should be considered separately. This is coincident with findings that when distinct genes are present between samples with and without metastasis, they can also reflect differences between the HTB56 and A549 cell lines. Conversely, in contrast to the high correlation of PC2 for gene expression and promoter methylation, correlations between gene expression and promoter methylation of individual genes were not significant (Fig S1). This might be because of the too small contribution of PC2s.

**Table 1:** Cancer disease association with genes selected in the present study based on Gendoo server.

Gene Symbol	RefSeq mRNA	Cancer associations (P-value)
		**PC2 vs PC2**

*SLC22A3*	NM_021977	Gonadoblastoma (0.0002), Dysgerminoma (0.00075), Testicular Neoplasms (0.00456), Ovarian Neoplasms (0.0297), Cell Transformation, Neoplastic (0.0384)

*DFNA5*	NM_004403	Melanoma (0.006),

*SPG20*	NM_015087	Hepatoblastoma (0.0033), Liver Neoplasms (0.00496)

*CYP1B1*	NM_000104	Breast Neoplasms (1.13 × 10^-45^), Endometrial Neoplasms (2.44 × 10^-12^), Lung Neoplasms (1.56 × 10^-9^), Prostatic Neoplasms (4.65e-9), Adenocarcinoma (6.03 × 10^-6^), Ovarian Neoplasms (1.35 × 10^-5^) Carcinoma, Squamous Cell (0.00018), Colorectal Neoplasms (0.000337), Head and Neck Neoplasms (0.00052), Adenoma, Liver Cell (0.0072), Urinary Bladder Neoplasms (0.012), Neoplasms (0.019), Carcinoma, Small Cell (0.028), Carcinoma, Non-Small-Cell Lung (0.0326)

*ALX1*	NM_006982	Carcinoma (0.000305), Chondrosarcoma (0.00129), Bone Neoplasms (0.0106), Uterine Cervical Neoplasms (0.011)

*TFPI2*	NM006528	Uterine Neoplasms (2.6 × 10^-21^), Neoplasm Invasiveness (1.18 × 10^-14^), Choriocarcinoma (2.33 × 10^-13^), Fibrosarcoma (7.98 × 10^-9^), Glioma (2.50 × 10^-8^), Cystadenocarcinoma (1.68 × 10^-5^), Lung Neoplasms (6.74 × 10^-5^), Carcinoma, Non-Small-Cell Lung (0.00559)

*HOXA9*	NM_152739	Leukemia, Myeloid (2.0 × 10^-48^), Leukemia, Myeloid, Acute (9.24 × 10^-30^), Cell Transformation, Neoplastic (4.64 × 10^-29^), Leukemia (9.46 × 10^-19^), Leukemia, Myelogenous, Chronic, BCR-ABL Positive (2.64 × 10^-14^), Precursor Cell Lymphoblastic Leukemia-Lymphoma (2.46 × 10^-8^), Precursor B-Cell Lymphoblastic Leukemia-Lymphoma (1.65 × 10^-6^), Myoma (0.00046), Leukemia, T-Cell (0.0012), Endodermal Sinus Tumor (0.0079), Seminoma (0.0157),

*HOXA11*	NM_005523	Uterine Neoplasms (8.23 × 10^-7^), Choriocarcinoma (3.97 × 10^-5^), Carcinoma, Endometrioid (0.0065), Adenocarcinoma, Clear Cell (0.00662), Wilms Tumor (0.0076),

*PCSK1*	NM000439	Bronchial Neoplasms (0.0022), Adenoma (0.0030), Adenoma, Islet Cell (0.0035), Bile Duct Neoplasms (0.011)

*SPARC*	NM_003118	Neoplasm Invasiveness (8.42 × 10^-14^), Glioma (1.35 × 10^-8^), Brain Neoplasms (1.01 × 10^-7^), Melanoma (2.99 × 10^-7^), Lung Neoplasms (1.43 × 10^-5^), Carcinoma (0.00013), Carcinoma, Non-Small-Cell Lung (0.0009)

		**PC3 vs PC3**

*HOXB2*	NM_002145	Lung Neoplasms (0.000159), Leukemia, Myeloid (0.000326), Pulmonary Emphysema (0.00139), Carcinoma, Embryonal (0.0025), Adenocarcinoma (0.0054), Leukemia, Erythroblastic, Acute (0.0096), Leukemia, Promyelocytic, Acute (0.0124), Carcinoma, Small Cell (0.0148), Carcinoma, Non-Small-Cell Lung (0.0387)

*CCDC8*	NM_032040	

*ZNF114*	NM_153608	

*DIO2*	NM_000793	Choriocarcinoma (0.000616), Carcinoma, Papillary (0.00366), Hemangioma (0.0099), Adenoma (0.019), Neuroblastoma (0.025)

*LAPTM5*	NM_006762	Carcinoma, Hepatocellular (0.000396), Liver Neoplasms (0.000495), Multiple Myeloma (0.00947), Neoplasm Recurrence (0.010), Cell Transformation, Neoplastic (0.032)

*RGS1*	NM_002922	Burkitt Lymphoma (3.55 × 10^-5^), Lymphoma, B-Cell (9.14 × 10^-5^), Leukemia-Lymphoma, Adult T-Cell (0.0076), Lymphatic Metastasis (0.0329), Skin Neoplasms (0.0364), Stomach Neoplasms (0.0454), Melanoma (0.0455)

*B3GALNT1*	NM_003781	Neuroblastoma (0.0034)

		**PC5 vs PC4**

*TINAGL1*	NM_022164	Carcinoma, Hepatocellular (0.000119), Neoplasms (0.0295)

*PMEPA1*	NM_020182	Prostatic Neoplasms (2.30e-12), Carcinoma, Renal Cell (0.0233), Kidney Neoplasms (0.032)

*CX3CL1*	NM_002996	Neuroblastoma (0.0014)

*ICAM1*	NM_000201	Melanoma (0.00305), astrocytoma (0.00644), Granular Cell Tumor (0.0166), Colonic Neoplasms (0.0233), Lymphoma, AIDS-Related (0.023), Adenoma, Oxyphilic (0.0433)

### The third PCs distinguish differences between samples with and without metastasis for HTB56 but not for A549

Because no PCs reflected differences between samples with and without metastasis, we considered additional PCs. Figure 2 shows the contributions of samples to the third PC (PC3). Although PC3s have even smaller contributions (0.2% for gene expression and 1.5% for promoter methylation) than PC1s or PC2s (Figure 3) their correlation is high. Thus, genes associated with PC3 represent differences between samples with and without metastasis and we finally identified a useful PC. Interestingly, PC3 exhibited differences between samples with and without metastasis only for the HTB56 cell line. However, since the two cell lines are distinct in terms of their oncogenic potential, it is not surprising that genes that exhibit differences between samples with and without metastasis for HTB56 did not exhibit differences between samples with and without metastasis for A549. Thus, we further applied integrated analysis using PCA based unsupervised FE. Selected genes are shown in Table 1. Figure S2 (Additional file 2) shows gene expression and promoter methylation of the selected genes. Again, whether genes selected based on mRNA expression and those based on promoter methylation were significantly overlapped was analyzed and *P*-values attributed to selected genes common between gene expression and promoter methylation were 3.5×10^-5 ^and 5.1×10^-4^. Thus, integrated analysis using PCA based unsupervised FE successfully identified genes with both aberrant mRNA expression and promoter methylation. The association of cancer disease and the selected genes were investigated by the Gendoo server, and the results are shown in Table 1. As expected, most of the selected genes were significantly associated with cancer disease. Correlations between gene expression and promoter methylation of individual genes were not significant (Fig. S2).

### The fourth PC of promoter methylation and the fifth PC of gene expression represent differences between samples with and without metastasis for A549 but not for HTB56

We further sought PCs that exhibited differences between samples with and without metastasis for A549. The fourth PC (PC4) of promoter methylation and the fifth PC (PC5) of gene expression demonstrated differences between samples with and without metastasis for the A549 cell line (Figures 2 and 3). Because the correlation between PC4 and PC5 were very high despite their small contributions (0.6% for PC4 of promoter methylation and 0.09% for PC5 of gene expression), integrated analysis using PCA based unsupervised FE could still be used. Selected genes are shown in Table 1. Figure S3 (Additional file 3) shows gene expression and promoter methylation of the individual genes.

*P*-values attributed to selected genes common between gene expression and promoter methylation were 9.8×10^-8^. Thus, integrated analysis using PCA based unsupervised FE was successful. Cancer diseases associated with the selected genes are listed in Table 1, and more than 50% were reported to be associated with cancer-related diseases. However, correlations between gene expression and promoter methylation of individual genes were not significant (Fig. S3).

## Discussion

Although the association of cancer-related disease and the selected genes were annotated by the Gendoo server, more detailed information, regarding whether genes are expressed or repressed in cancers, will be useful. In addition, since the Gendoo server was last updated in April 2012, recent information might be missing. To fill these gaps, we will discuss the selected genes individually citing actual studies.

### HOXB2

*HOXB2 *has a Homeobox domain in the central region. Figure S4 (Additional file 4) shows the tertiary structure of the homeobox domain in *HOXB2 *predicted by FAMS. The homeodomain fold is a protein structural domain that binds to DNA or RNA and is commonly found in transcription factors. *HOXB2 *was upregulated in pancreatic cancer [12] as a part of the retinoic acid (RA) signaling pathway, which is generally regarded to be a potential anti-tumor agent [13]. *HOXB2 *also promotes the invasion of lung cancer cells by regulating metastasis-related genes [14]. Considering these studies, it was not surprising that *HOXB2 *might also have a critical role in NSCLC.

### CCDC8

Neither FAMS nor phyre2 predicted a significant tertiary structure for *CCDC8*, which is reported to be a cofactor required for p53-mediated apoptosis through interactions with *OBSL1 *[15]. Thus, because p53 protein is a typical tumor suppressor, it is likely that *CCDC8 *has a critical role in NSCLC.

### ZNF114

*ZNF114 *has one KRAB box and four Zinc-finger double domains. Figure S5 (Additional file 4) shows the Zinc-finger domains as predicted by FAMS. Since the Zinc-finger double domain functions in DNA binding, *ZNF114 *might also be a DNA binding protein. KRAB is a transcription repression domain, thus *ZNF114 *might be a transcription suppressor. Unfortunately, very few studies of *ZNF114 *have been published. However, mutation of CTCF that has seven Zinc-finger double domains was reported to be associated with tumors [16]. GC79 that has multiple Zinc-finger double domains was reported to be associated with apoptosis of prostate cancer cells [17]. Studies related to proteins with Zinc-finger double domains indicate *ZNF114 *might also have a role in NSCLC.

### DIO2

Figure S6 (Additional file 4) shows the tertiary structure of *DIO2 *as predicted by FAMS. *DIO2 *belongs to the iodothyronine deiodinase family and is underexpressed in benign and malignant thyroid tumors [18]. *DIO2 *expression was also shown to be higher in most brain tumors [19]. Thus, although it is unclear whether *DIO2 *is generally oncogenic or tumor suppressive, it appears to be related to cancer. Therefore, *DIO2 *is likely to be related to NSCLC.

### LAPTM5

Neither FAMS nor phyre2 predicted a significant tertiary structure for *LAPTM5*, a transmembrane protein that was reported to be associated with spontaneous regression of neuroblastomas [20]. Inactivation of the *E3/LAPTM5 *gene by chromosomal rearrangement and DNA methylation in human multiple myeloma was observed [21]. Expression of *LAPTM5 *was also elevated in human B lymphomas [22]. Although there have been no reports indicating a relationship between *LAPTM5 *and NSCLC, *LAPTM5 *might have a critical role in NSCLC.

### RGS1

*RGS1 *contains a regulator of G protein signaling domain. The tertiary structure of *RGS1 *is available in the Protein Data Bank (PDB) (Fig. S7 in Additional file 4). Regulator of G-protein signaling (RGS) proteins are related to cancer biology [23] and genetic variations in these genes are associated with survival in late-stage NSCLC [24]. *RGS1 *was overexpressed in a gene expression-profiling study of melanoma [25]. RGS is thought to be related to the functionality of G protein-coupled receptors [26] that are often drug targets. Thus, RGS might be a promising drug target candidate for therapy of NSCLC.

### B3GALNT1

*B3GALNT1 *is a galactosyl transferase that catalyzes the transfer of galactose. Fig. S8 (Additional file 4) shows the tertiary structure of *B3GALNT1 *as predicted by FAMS. Numerous studies have suggested a relationship between galactosyl transferase and cancer, including the use of galactosyl transferase as a tumor biomarker for ovarian clear cell carcinoma [27,28]. Alternatively, cancer-associated isoenzymes of serum galactosyl transferase were reported in various cancers [29]. Thus, *B3GALNT1 *might have a role in NSCLC progression, although no reports have demonstrated a specific relationship between *B3GALNT1 *and cancer.

### TINAGL1

*TINAGL1 *is papain family cysteine protease that degrades proteins. Figure S9 (Additional file 4) shows the tertiary structure as predicted by FAMS. *TINAGL1 *is a Sec23a-dependent metastasis suppressor [30] and was reported to be upregulated in highly metastatic tumors [31]. Thus, it was reasonable that it was selected as a cancer-related gene candidate by our methodology.

### PMEPA1

Neither FAMS nor phyre2 predicted a significant tertiary structure for *PMEPA1*, a transmembrane prostate androgen-induced protein that enhances tumorigenic activity in lung cancer cells [32]. It was also reported to be upregulated in ovarian cancer [33], colon cancer [34] and renal cell carcinoma [35]. Considering these studies, *PMEPA1 *was reasonably selected as a NSCLC-related gene by PCA based unsupervised feature extraction (FE) mediated integrated analysis.

### CX3CL1

*CX3CL1 *contains a small cytokine (intecrine/chemokine) interleukin-8 (IL-8)-like domain. Figure S10 (Additional file 4) shows the tertiary structure of the IL-8 domain as predicted by FAMS. The IL-8 pathway was reported to be important in cancer [36] and *CX3CL1 *expression was associated with a poor outcome in breast cancer patients [37] as it promotes breast cancer via transactivation of the epidermal growth factor pathway [38]. A complex role for *CX3CL1 *in cancer has been reported [39]. Thus, it was reasonable that *CX3CL1 *was selected by our methodology.

### ICAM1

Intercellular adhesion molecule (ICAM) contains an N-terminal domain and three immunoglobulin domains. The tertiary structure of *ICAM1 *was available in PDB (Fig. S11 in Additional file 4). Many studies have reported a relationship between *ICAM1 *and cancer. *ICAM1 *expression was reported to determine the malignant potential of cancer [40], to have a role in the invasion of human breast cancer cells [41], and upregulated endogenous ICAM-1 reduced ovarian cancer cell growth in the absence of immune cells [42]. Thus, it is reasonable that *ICAM1 *was selected as a potential NSCLC therapy target by our methodology.

### *TINAGL1 *as a drug target gene candidate

In this study, we selected multiple genes that might be involved in the progression of NSCLC metastasis. Most of selected genes are potential cancer-related genes. Thus, it is reasonable to regard these genes as therapy targets. Among those selected, we investigated *TINAGL1 *as a potential drug target gene, because although *TINAGL1 *is regarded to be a tumor suppressor, in this study it was upregulated in a metastasis-enhanced cell line. Naba et al [31] also reported that *TINAGL1 *was up-regulated in highly metastatic tumors. Thus, inhibition of *TINAGL1 *might be a potential therapeutic target for the treatment of metastatic NCSLC. Furthermore, although we used a profile based drug discovery software, chooseLD (Insilico Science Co., Tokyo, Japan) [43], for *in silico *drug screening, it required the tertiary structure of the target protein and multiple ligand compounds whose binding structure to the protein were known. *TINAGL1 *satisfied these requirements as follows. To infer the tertiary structure of *TINAGL1*, we uploaded the amino acid sequence NP_071447.1 retrieved from RefSeq to FAMS and phyre2. Because there was no significant difference between tertiary structures inferred by FAMS and phyre2, hereafter we used the structure inferred by FAMS.

Based on FAMS, *TINAGL1 *has many tertiary structures registered in PDB that can be used for tertiary structure predictions. Among them, the "A" chain of PDB ID: 2DCC (2DCC_A) has a 32% sequence similarity with *TINAGL1 *and is accompanied by multiple highly similar (> 95% sequence similarity) tertiary structures registered in PDB (PDB ID: 1IT0_A, 1QDQ_A, 2DC6_A, 2DC7_a, 2DC8_A, 2DC9_A, 2DCA_A, and 2DCD_A). Because all of these structures have more than one ligand that binds to protein, we had a large number of ligand-protein binding structures that could be used for *in silico *drug screening using chooseLD. We selected 2DCC_A, a protein structure of *TINAGL1 *from aa 204 to 455 for modeling by FAMS. Because 2DCC_A is cathepsin, hereafter we called this structure the cathepsin domain. To confirm that chooseLD could predict ligand binding to the cathepsin domain, we attempted to identify a known ligand that binds to the cathepsin domain. ChEMBL [44] was identified by a BLAST search using the cathepsin domain amino acid sequence. Thus, *Plasmodium falciparum *3D7 (CHEMBL1250370), a putative protease, was found to have 47.22% sequence similarity with the cathepsin domain. There were five assay experiments for this protein. Among them, CHEMBL1244076 was employed to list candidate binding ligands. Three ligands were reported to inhibit *Plasmodium falciparum *3D7. Among them, CHEMBL1242746 and CHEMBL1242747 (Fig. S12 in Additional file 4) were employed as potential binding ligands to *TINAGL1*. Then chooseLD was used to test the two ligands using 15 template ligand proteins 3S3Q_C1P, 3S3R_0IW_00, 3AI8_HNQ_01, 1GMY_hld_00, 2DCC_77B, 1ITO_E6C, 1QDQ_074, 2DC6_73V, 2DC7_042, 2DC8_59A, 2DC9_74M, 2DCA_75V, 2DCB_76V, 2DCD_78A, and 3PDF_LXV. Fig. S13 (Additional file 4) shows the binding of CHEMBL1242565 and CHEMBL514348 to *TINAGL1 *(Additional file 5 for template ligand binding to *TINAGL1*).

Binding affinity of ligands to *TINAGL1 *was evaluated by Cyscore [45] (Table 2). All template ligands had negative, thus better, Cyscores. Although CHEMBL1242746 or CHEMBL1242747 did not achieve a negative Cyscore, CHEMBL1242747 had a low positive Cyscore that was lower than the lowest template ligands. If we consider these two were only reported to bind to proteins with a *TINAGL1 *sequence similarity of 45%, the Cyscore attributed to CHEMBL1242746 or CHEMBL1242747 was good and demonstrated the ability of chooseLD to infer proper binding configurations of potential ligands. Since chooseLD inferred suitable binding modes for two potential ligands, we concluded that *in silico *drug discovery is possible for *TINAGL1*, which might be a promising candidate drug target.

**Table 2:** Inference of ligand binding affinity to *TINAGL1*.

	Hydrophobic	Vdw	HBond	Ent	Cyscore
1GMY_hld	-0.4156	-1.77	0	0.378	-1.8086
1ITO_E6C	-0.3024	-0.56	0	0.42	-0.4461
1QDQ_074	-1.3611	-1.88	0	0.42	-2.8243
2DC6_73V	-0.552	-1.79	0	0.63	-1.7081
2DC7_042	-0.5374	-1.56	0	0.588	-1.514
2DC8_59A	-0.5766	-1.6	0	0.462	-1.717
2DC9_74M	-1.3254	-1.24	0	0.462	-2.1026
2DCA_75V	-1.1413	-1.52	0	0.546	-2.1103
2DCB_76V	-1.6095	-1.76	0	0.63	-2.7419
2DCC_77B	-0.5932	-2.07	0	0.63	-2.0373
2DCD_78A	-1.5344	-1.65	0	0.42	-2.7627
3AI8_HNQ	-0.7476	0.635	0	0.042	-0.0704
3PDF_LXV	-0.6498	-0.96	0	0.168	-1.4434
3S3Q_C1P	-1.2913	-1.61	0	0.504	-2.4019
3S3R_0IW	-1.0358	-0.97	0	0.504	-1.5028
CHEMBL1242746	-1.3699	2.768	0	0.42	1.8177
CHEMBL1242747	-1.561	1.431	0	0.336	0.206

Cyscore and Hydrophobic, Vdw (van der Waals), Hbond (hydrogen bond), and Ent (entropy) computed by Cyscore programs of 15 template ligands and two drug candidate compounds.

### *B3GALNT1 *as a candidate drug target gene

Another potential drug target gene is *B3GALNT1*. Substrates such as UDP-galactose and UDP-N-acetylglucosamine bind to *B3GALNT1 *and after various catalytic reactions, UDP remains bound to *B3GALNT1*. Thus, if compounds that inhibit UDP binding to *B3GALNT1 *that compete with UDP can be identified, the function of *B3GALNT1 *can be inhibited. *B3GALNT1 *is a galactosyl transferase, which is often reported to be upregulated in various cancers. Thus, inhibition of *B3CALNT1 *might be a potential therapeutic target for NSCLC. Fig. S14 (Additional file 4) shows the UDP and UDP-N-acetylglucosamine binding to *B3GALNT1 *predicted by chooseLD. Because both bind to the same pockets of *B3GALNT1*, chooseLD can be used to identify other compounds that bind to and inhibit *B3GALNT1*.

### Inconsistency between gene expression and promoter methylation of individual genes

Although Figs. S1, S2 and S3 show the gene expression and promoter methylation of individual genes associated with selected PCs, coincidence between gene expression and promoter methylation is relatively poor. Gene selection was reliable because *P*-values attributed to the simultaneous selection of genes for gene expression and promoter methylation PCs were significant and the selected genes were associated with cancer-related genes (Table 1). To resolve the discrepancy between the significant selection of genes and poor coincidence of individual genes between gene expression and promoter methylation, we considered promoter methylation by sequencing technology, which was performed simultaneously with microarray measurements. Figure S15 (Additional file 6) shows the promoter methylation profile of selected genes by sequencing technology. Although measurements were unfortunately not available for all observations, promoter methylation measured by sequencing technology was more coincident (negatively correlated) with gene expression than by microarray. Since sequencing technology is more reliable than microarray, poor consistency between gene expression and promoter methylation might be explained by the poor ability of microarray to measure promoter methylation. Thus, discrepancies are expected to be resolved when promoter methylation is measured with high accuracy.

### Superiority and novelty of the proposed method

The novel method employed in the current study has a number of advantages compared with existing conventional methodologies. To demonstrate failure of the conventional approaches, first we tried detect genes that have a negative correlation between mRNA expression and promoter methylation. Pairs of mRNA expression microarray probes and promoter methylation probes to which common mRNA RefSeq IDs are attributed were collected. Then Pearson correlation coefficients were computed for all pairs as in Figure 3. The obtained *P*-values were adjusted by Benjamin-Hochberg criterion since there were more than twenty thousand pairs. Only one gene had an adjusted *P*-value <0.05. This clearly demonstrates the usefulness of applying PCA, without which almost no significant correlations would be detected.

Next, we used the t-test to detect significant differences between samples with and without metastasis. The t-test was applied to all probes and obtained *P*-values were again adjusted by Benjamin-Hochberg criterion to suppress failed positives because of high numbers of observations. For comparison between A549 cell lines with and without metastasis, no probes had adjusted *P*-values <0.05 for either mRNA expression or promoter methylation. For comparison between HTB56 cell lines with and without metastasis, although as many as 434 probes had adjusted *P*-values <0.05 for mRNA expression, there were no probes for promoter methylation. This also suggests the usefulness of applying PCA without which no significant aberrant promoter methylation or mRNA expression would be detected.

These difficulties for the detections of significant correlations and aberrant mRNA expression/promoter methylation were caused by the small number of replicates in the study (three replicates for each mRNA expression and two replicates for each promoter methylation). Since PCA can detect the behavior of a group of probes, this difficulty can be compensated for and explains why only applying PCA can detect significant outcomes.

Finally, we would like to emphasize some of the novelties of the PCA based methodology. Although PCA itself is a frequently used method, the current study applied PCA differently from conventional methodology. First, PCA is usually used to embed samples into low dimensional space to demonstrate the groupings of samples, while this study used embedded probes. This enabled the identification of what each PC discriminates as in Figure 2. To our knowledge, PCA has rarely been used this way. Second, we did not ignore PCs to which only tiny contributions were attributed (PC2, PC3, PC4 and PC5 investigated in this study had at most 10% contributions), while standard procedures recommend ignoring such PCs since it is impossible to distinguish them from background noise. The reason why such small a contribution can have meaning is because of the huge number of probes used. Since as many as twenty to thirty thousand probes are analyzed, contributions as little as 0.1% can correspond to several tens of probes. This is a new concept, and thus it is not generally recognized that small contributions can have meaning. Therefore, although the usage of PCA itself is not novel, the method used in this study is new.

### Comparison with tissue samples associated with metastasis

Although we detected many significant results in this study, the original observation of the analysis was cell lines. Since cell lines have a greater tendency to exhibit clear outcomes than tissues, it is unclear whether the study results could be observed using more relevant samples, such as tissues. To confirm this, we consulted a database that stores the expression of tissues with and without metastasis. Unfortunately, we could not find a data set where NSCLC tissues were treated. Thus, we compared melanoma with and without metastasis (GEO ID: GDS3966 [46]). Because metastasis is commonly caused by similar factors, e.g, the loss of cell adhesion, independent of the type of cancer, comparison of melanoma with and without metastasis was expected to share aberrant mRNA expression with NSCLC with and without metastasis (Table 3). Although not all eleven genes were observed, among seven of the genes included, six had a *P*-vales <0.05 by Kolmogorov-Smirnov (KS) test. The KS test was used because it is more robust (non-metric) than the t-test, thus was more suitable for samples with a high background noise such as tissues. The results shown in Table 3 suggest that the findings in this cell line might also be observed in tissue samples.

**Table 3:** *P*-values that represent significant differences of melanoma tissue samples between those with and without metastasis.

Gene	Metastasis > no metastasis	No metastasis >metastasis
*HOXB2*	**3 × 10^-3^**	1
*LAPTM5*	**5 × 10^-2^**	8 × 10^-1^
*RGS1*	1 × 10^-1^	**4 × 10^-2^**
*TINAGL1*	9 × 10^-1^	**3 × 10^-2^**
*PMEPA1*	6 × 10^-1^	**5 × 10^-3^**
*CX3CL1*	1	1 × 10^-1^
*ICAM1*	**3 × 10^-3^**	1

Values were computed using the Kolmogorov-Smirnov test. *P*-values <0.05 highlighted in bold.

### Transcription factor aryl hydrocarbon receptor targets selected eleven genes

Although the biological significance of individual genes was confirmed, it would be more useful if biological reasons for the commonality between the genes could be identified. We uploaded mRNA RefSeq IDs for eleven genes to DAVID [47] and found that all eleven genes were targets of the transcription factor aryl hydrocarbon receptor (AHR), reported to be primary factor that causes lung cancer [48]. AHR was also suggested to promote metastasis [48]. Given that promoter methylation is primarily related to transcription factors binding to promoters, it is reasonable that AHR was identified by the integrated analysis of mRNA expression and promoter methylation. This also suggests that our methodology and analysis are suitable for identification of potential cancer causing genes for NSCLC.

## Conclusions

This study performed the integrated analysis of promoter methylation and gene expression using PCA based unsupervised FE. It selected eleven genes that were differently expressed and which had different promoter methylation patterns between cell lines with and without metastasis ability. *P*-values attributed to the simultaneous selection between gene expression and promoter methylation were significant and many cancer-related diseases were associated with the eleven genes selected. Two of the eleven genes selected, *B3GALNT1 *and *TINAGL1*, were identified as drug target candidates that might suppress metastasis in NSCLC. Further detailed and advanced studies are required to confirm these findings.

## Methods

### Promoter methylation and gene expression profiles

Promoter methylation profiles were downloaded from Gene Expression Omnibus (GEO) with GEO ID: GSE52144 that included two replicates of HTB56 cell lines with (H3R_d0) and without (H0R_d0) metastasis ability and A549 cell lines with (A3R_d0) and without (A0R_d0) metastasis ability. Gene expression profiles were downloaded from GEO with GEO ID: GSE52143 that included three replicates of the samples in GSE52144. For these two cell lines, data sets deposited in the "Series Matrix Files" were retrieved. Promoter methylation measured by sequencing was obtained from GEO with GEO ID: GSE52140. Within GSE52140_RAW.tar, eight files corresponding to those in GSE52144, (two replicates of H0R_d0. H3R_d0, A0R_d0 and A3R_d3) were used.

### Retrieval of promoter methylation from sequencing data

Information of genes annotated by RefSeq mRNA, (transcription start and end sites, strand, chromosome name) in hg19 human genome were retrieved using the Table browser of the Genome Browser [49]. Using this information, methylation sites between 1500 bps upstream and 500 bps of transcription start sites were collected. Mean β-values, (the ratio of methylated sites among the total number of methylation sites), were employed as promoter methylation for each RefSeq gene.

### Integrated analysis of gene expression and promoter methylation using PCA based unsupervised FE

First, PCA was applied to gene expression and promoter methylation and each probe was embedded into a two dimensional space spanned with the first and the second PC scores. Then contributions of each probe to each PC were investigated and biologically meaningful PCs were selected. The 100 top outlier probes with larger (positively larger) or smaller (negatively larger) PC scores were extracted for each PC. The coincidence between selected probes for gene expression and promoter methylation was estimated as follows. If contributions of each probe to PCs were positively correlated between gene expression and promoter methylation, then intersections between gene expression outlier probes having larger (smaller) PC scores and promoter methylation outlier probes having smaller (larger) PC scores were sought, since gene expression and promoter methylation were expected to be negatively correlated with each other. Conversely, if contributions of each probe to PCs were negatively correlated between gene expression and promoter methylation, intersections between gene expression outlier probes having larger (smaller) PC scores and promoter methylation outlier probes having larger (smaller) PC scores were sought. *P*-values attributed to simultaneous selection of probes between gene expression and promoter methylation were computed by distribution that obeyed binomial distribution as follows:

1-P(x,100,100/y)

where × is the number of commonly selected probes between the top 100 outliers of gene expression and promoter methylation, y is total number of probes on the microarray, and P is the cumulative frequency of binomial distribution.

### Cancer-related disease association of selected genes by Gendoo server

Cancer-related disease association was identified using the Gendoo server. RefSeq mRNA was transformed to a gene symbol, which was uploaded to the Gendoo server with "human" as the target species. Among the associated diseases, those related to cancer and with *P*-values <0.05 are listed in Table 1.

### Tertiary protein structure prediction using profile based inference servers

Amino acid sequences retrieved from Uniprot (Additional file 7) were uploaded to two profile based tertiary structure databases, FAMS and phyre2. Because no significant differences were observed between the two databases, inferences by FAMS were used for all further analyses.

### Domain annotation by Pfam

To annotate domains included in each gene, we uploaded amino acid sequences to pfam [50], and determined the domains contained in each amino acid sequence.

### Ligand-protein docking inference by chooseLD

ChooseLD is a profile-based ligand-protein docking affinity evaluation software. ChooseLD requires well-predicted or observed tertiary structures of target genes and known binding configurations of multiple compounds to which drug candidate compounds can be aligned. For the *TINAGL1 *gene, there are 15 known ligands that bind *TINAGL1 *or highly similar proteins (> 95% sequence similarity). Thus, *in silico *drug discovery was performed for *TINAGL1*. However, UDP is the only ligand with a known configuration that can bind to *B3GALNT1*. Fortunately, *B3GALNT1 *can bind substrates in contrast to other proteins that only bind to other proteins, thus *in silico *drug discovery is easier since compounds that bind to *B3GALNT1 *by substituting UDP can be determined. Therefore, *TINAGL1 *and *B3GALNT1 *might be potential drug candidate genes.

### Ligand-protein docking affinity evaluation by Cyscore

**Figure 4:**
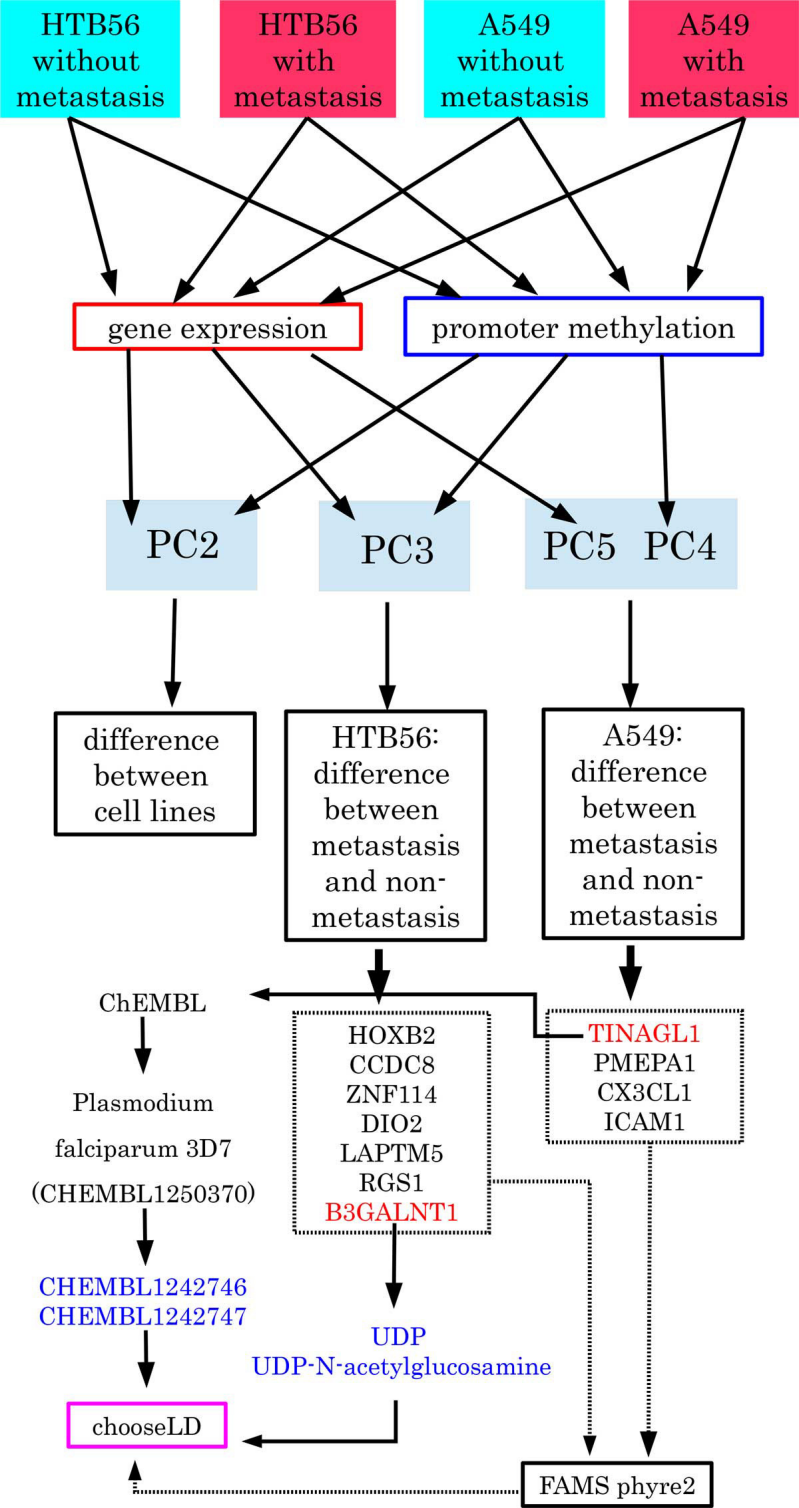
**Schematic figure of data processing**. Schematic of data processing.

Cyscore was used to evaluate binding affinity to *TINAGL1*, [45]. Hydrogen was added to PDB files of *TINAGL1 *by pymol [51] using the "h_add" command and was added to mol2 files of ligands by babel [52] using the "-h" option. Then, Cyscore was applied to the PDB file of *TINAGL1 *and mol2 files of ligands. Although pdb2pqr [53] (default settings) was used to add hydrogen to *TINAGL1*, the resulting Cyscore was not improved. Thus, we decided to use pymol. These processes are shown in Figure 4.

## Competing interests

The authors declare that they have no competing interests.

## Authors' contributions

HU performed *in silico *ligand-protein binding inference by chooseLD. MI predicted tertiary structures of proteins using FAMS. YHT designed and supervised the whole research project, performed integrated analysis, predicted tertiary structures of proteins by phyre2, obtained Cyscores and wrote the paper.

## Supplementary Material

Additional file 1 **Fig. S1 Gene expression and promoter methylation associated with PC2**. Gene expression and promoter methylation associated with PC2. Left column: gene expression, right column: promoter methylation. NM_021977 (SLC22A3), NM_004403 (DFNA5), NM_015087 (SPG20), NM_000104 (CYP1B1), NM_006982 (ALX1), NM_006528 (TFPI2), NM_152739 (HOXA9), NM_005523 (HOXA11 ), NM_000439 (PCSK1), NM_003118 (SPARC). (Black open circles: A549 without metastasis, red triangles: A549 with metastasis, green crosses: HTB56 without metastasis, blue crosses: HTB56 with metastasis). Left column: gene expression, right column: promoter methylation. "cor" indicates Pearson correlation coefficients between gene expression and promoter methylation averaged within each of four categories and "P" is attributed to "cor". Click here for file

Additional file 2 **Fig S2 Gene expression and promoter methylation associated with PC3**. Gene expression and promoter methylation associated with PC3. Left column: gene expression, right column: promoter methylation. NM_002145 (HOXB2), NM_032040 (CCDC8), NM_153608 (ZNF114), NM_000793 (DIO2), NM_006762 (LAPTM5), NM_002922 (RGS1), NM_003781 (B3GALNT1). (Black open circles: A549 without metastasis, red triangles: A549 with metastasis, green crosses: HTB56 without metastasis, blue crosses: HTB56 with metastasis). Left column: gene expression, right column: promoter methylation. "cor" indicates Pearson correlation coefficients between gene expression and promoter methylation averaged within each of four categories and "P" is attributed to "cor". Click here for file

Additional file 3 **Fig. S3 Gene expression and promoter methylation associated with PC4 (promoter methylation) and PC5 (gene expression)**. Gene expression and promoter methylation associated with PC4 (promoter methylation) and PC5 (gene expression). Left column: gene expression, right column: promoter methylation. NM_022164 (TINAGL1), NM_020182 (PMEPA1), NM_002996 (CX3CL1), NM_000201 (ICAM1). Contributions of samples (black open circles: A549 without metastasis, red triangles: A549 with metastasis, green crosses: HTB56 without metastasis, blue crosses: HTB56 with metastasis) to PCs. Left column: gene expression, right column: promoter methylation. "cor" indicates Pearson correlation coefficients between gene expression and promoter methylation averaged within each of four categories and "P" is attributed to "cor". Click here for file

Additional file 4 **Supplementary figures**. Supplementary figures from Fig. S4 to Fig. S14. Click here for file

Additional file 5 **Ligand binding configuration to *TINAGL1*. **Full list of ligands that bind to ***TINAGL1*.** Click here for file

Additional file 6 **Fig. S15 Promoter methylation profile of selected genes measured by sequencing technology**. Promoter methylation measured by sequencing, NM_002145 (***HOXB2***), NM_032040 (***CCDC8***), NM153608 (***ZNF114***), NM_006762 (***LAPTM5***), NM_003781 (***B3GALNT1***), NM_022164 (***TINAGL1***), NM_020182 (***PMEPA1***), NM_002996 (***CX3CL1***), and NM_000201 (***ICAM1***). Click here for file

Additional file 7 **Amino acid sequences used for tertiary protein structure prediction**. Amino acid sequences retrieved from Uniprot of selected genes (in fasta format). Click here for file

## Declarations

Publication costs for this article were funded by grant KAKENHI 26120528 from National Institute of Informatics, Japan.

This article has been published as part of *BMC Genomics *Volume 15 Supplement 9, 2014: Thirteenth International Conference on Bioinformatics (InCoB2014): Computational Biology. The full contents of the supplement are available online at http://www.biomedcentral.com/bmcgenomics/supplements/15/S9.
