# Supplementary material for: TINAGL1 and B3GALNT1 are potential therapy target genes to suppress metastasis in non-small cell lung cancer
Source: BMC Genomics. 2014 Dec 8;15(Suppl 9):S2. doi: 10.1186/1471-2164-15-S9-S2 (PMC4290609; doi:10.1186/1471-2164-15-S9-S2)
Supplement: Additional file 1 — Fig. S1 Gene expression and promoter methylation associated with PC2. Gene expression and promoter methylation associated with PC2. Left column: gene expression, right column: promoter methylation. NM_021977 (SLC22A3), NM_004403 (DFNA5), NM_015087 (SPG20), NM_000104 (CYP1B1), NM_006982 (ALX1), NM_006528 (TFPI2), NM_152739 (HOXA9), NM_005523 (HOXA11 ), NM_000439 (PCSK1), NM_003118 (SPARC). (Black open circles: A549 without metastasis, red triangles: A549 with metastasis, green crosses: HTB56 without metastasis, blue crosses: HTB56 with metastasis). Left column: gene expression, right column: promoter methylation. "cor" indicates Pearson correlation coefficients between gene expression and promoter methylation averaged within each of four categories and "P" is attributed to "cor". [file 1471-2164-15-S9-S2-S1.pdf]

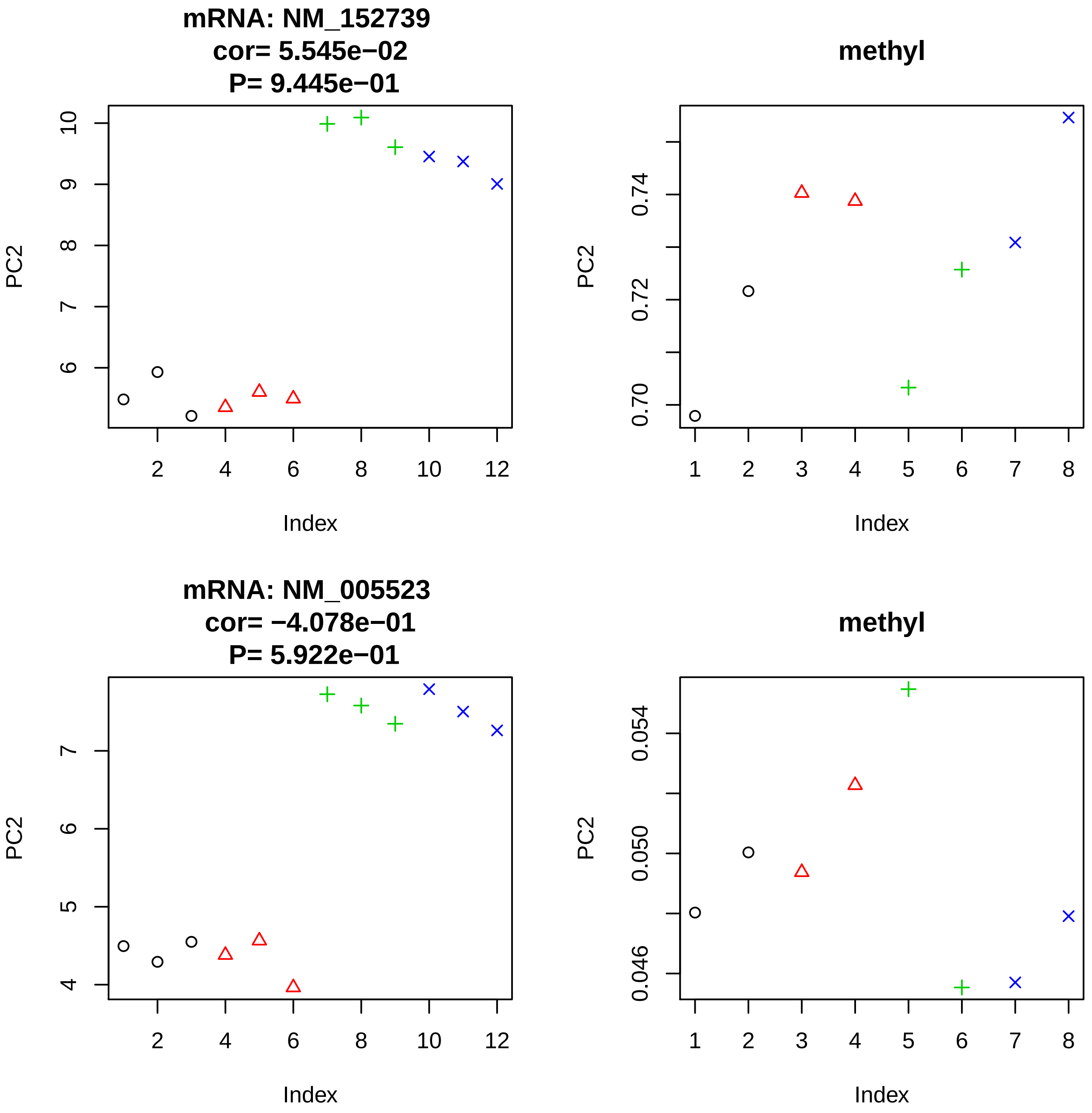
mRNA: NM_152739
cor= 5.545e−02
P= 9.445e−01
PC2
Index
methyl
PC2
Index
mRNA: NM_005523
cor= −4.078e−01
P= 5.922e−01
PC2
Index
methyl
PC2
Index

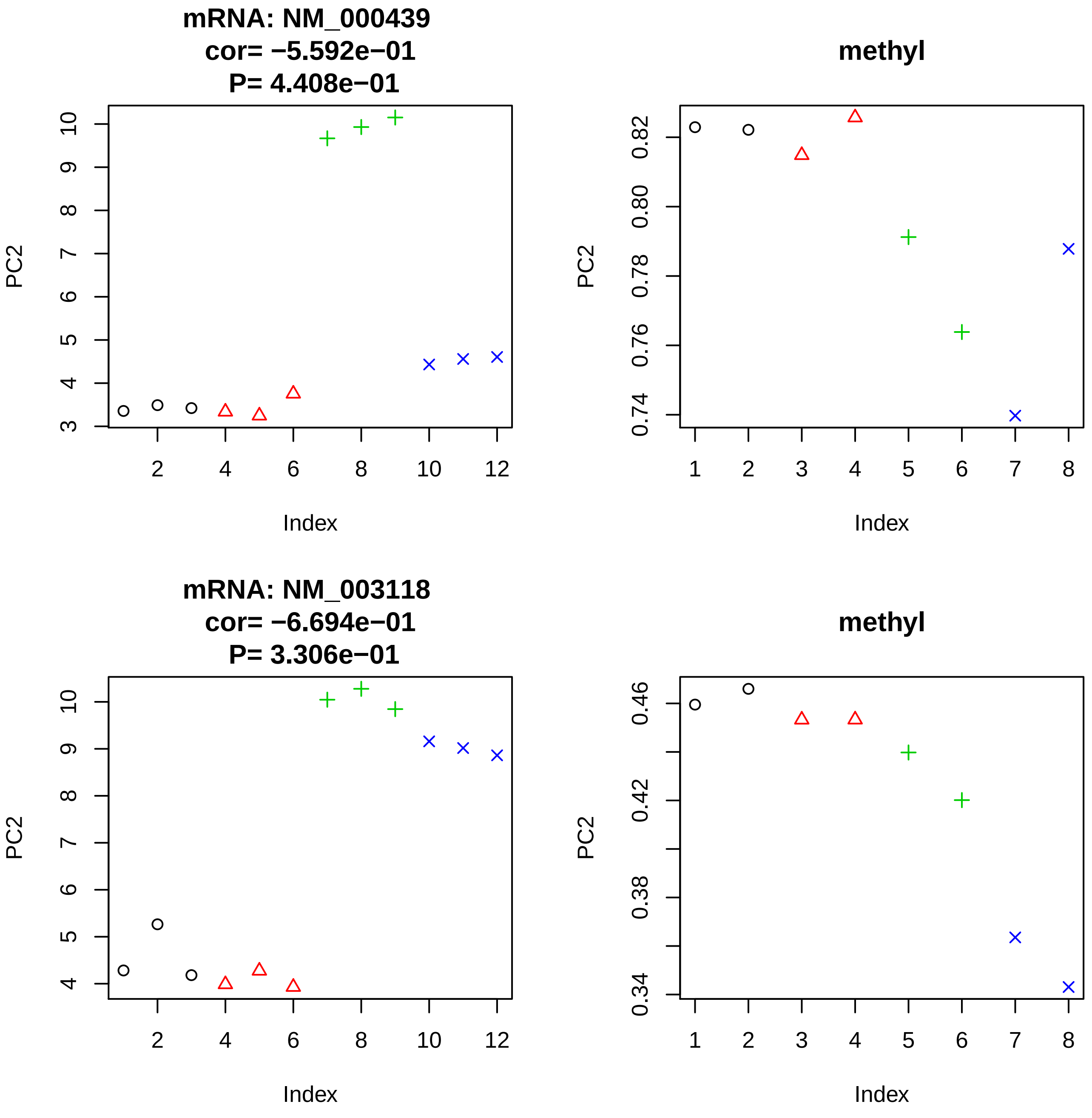
mRNA: NM_000439
cor= −5.592e−01
P= 4.408e−01
PC2
3
4
5
6
7
8
9
10
2
4
6
8
10
12
Index
methyl
PC2
0.74
0.76
0.78
0.80
0.82
1
2
3
4
5
6
7
8
Index
mRNA: NM_003118
cor= −6.694e−01
P= 3.306e−01
PC2
4
5
6
7
8
9
10
2
4
6
8
10
12
Index
methyl
PC2
0.34
0.38
0.42
0.46
1
2
3
4
5
6
7
8
Index

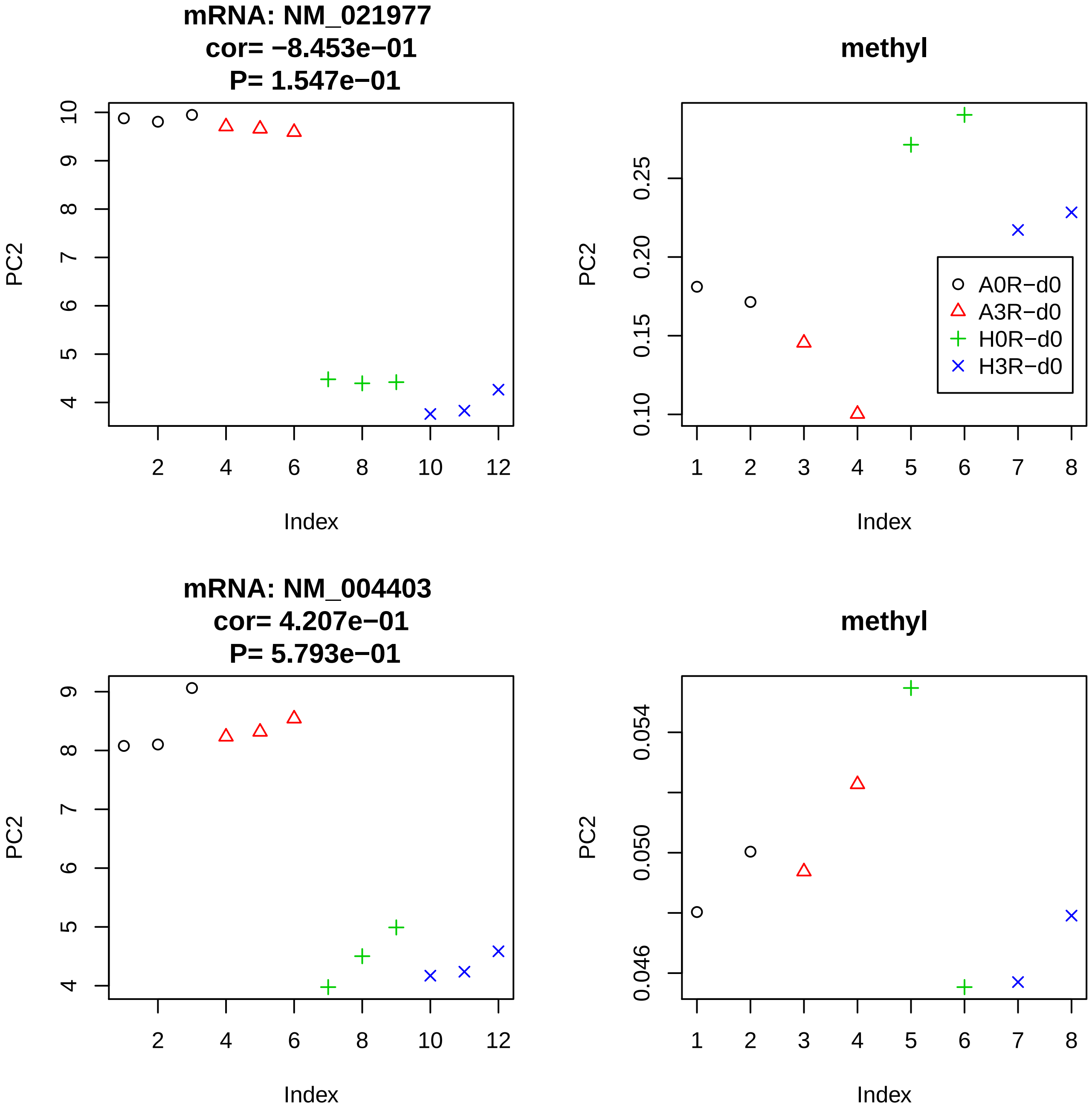

mRNA: NM_021977
cor= −8.453e−01
P= 1.547e−01
PC2
Index
methyl
PC2
Index
A0R−d0
A3R−d0
H0R−d0
H3R−d0
mRNA: NM_004403
cor= 4.207e−01
P= 5.793e−01
PC2
Index
methyl
PC2
Index

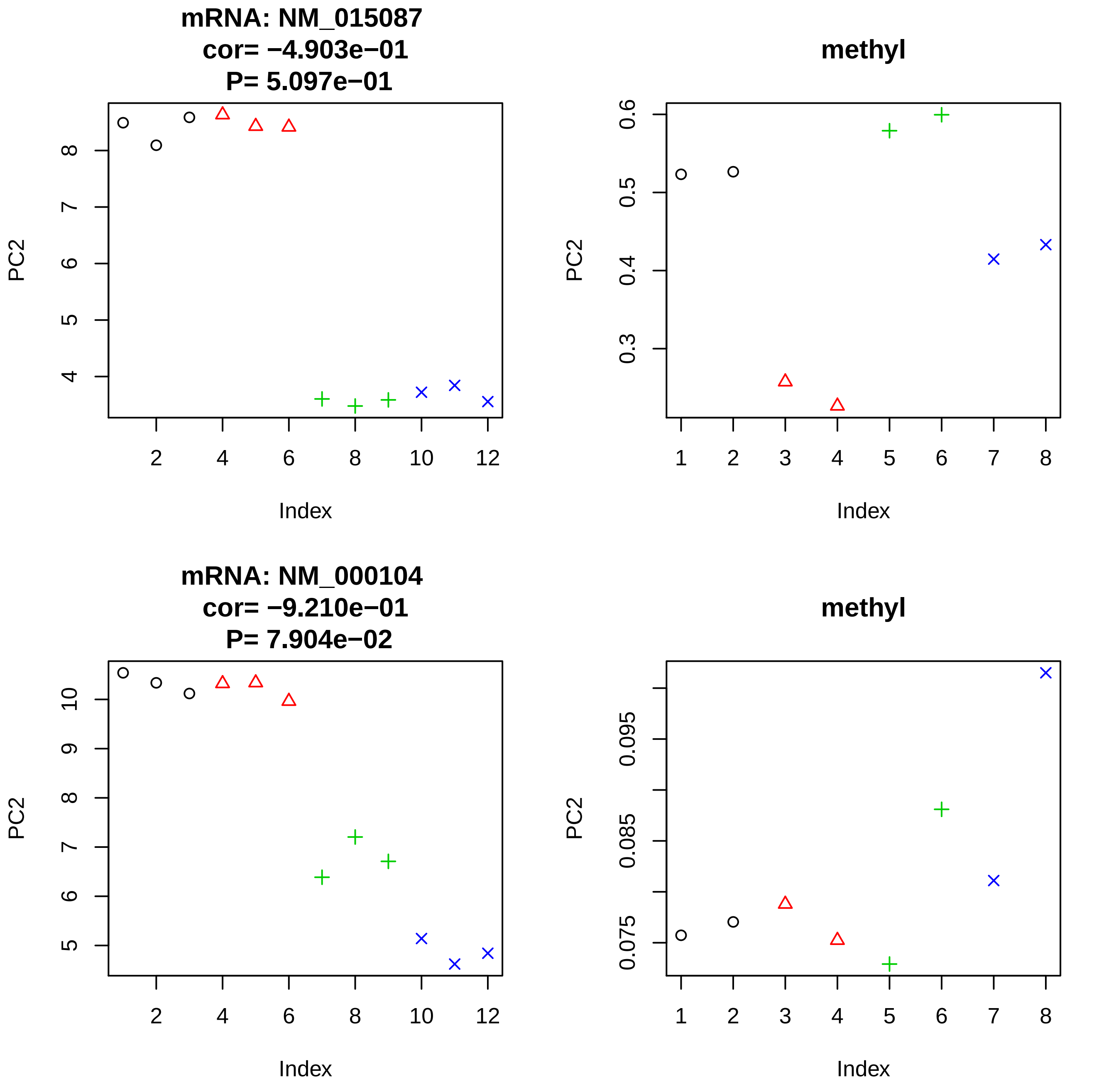
mRNA: NM_015087
cor= −4.903e−01
P= 5.097e−01
PC2
Index
methyl
PC2
Index
mRNA: NM_000104
cor= −9.210e−01
P= 7.904e−02
PC2
Index
methyl
PC2
Index

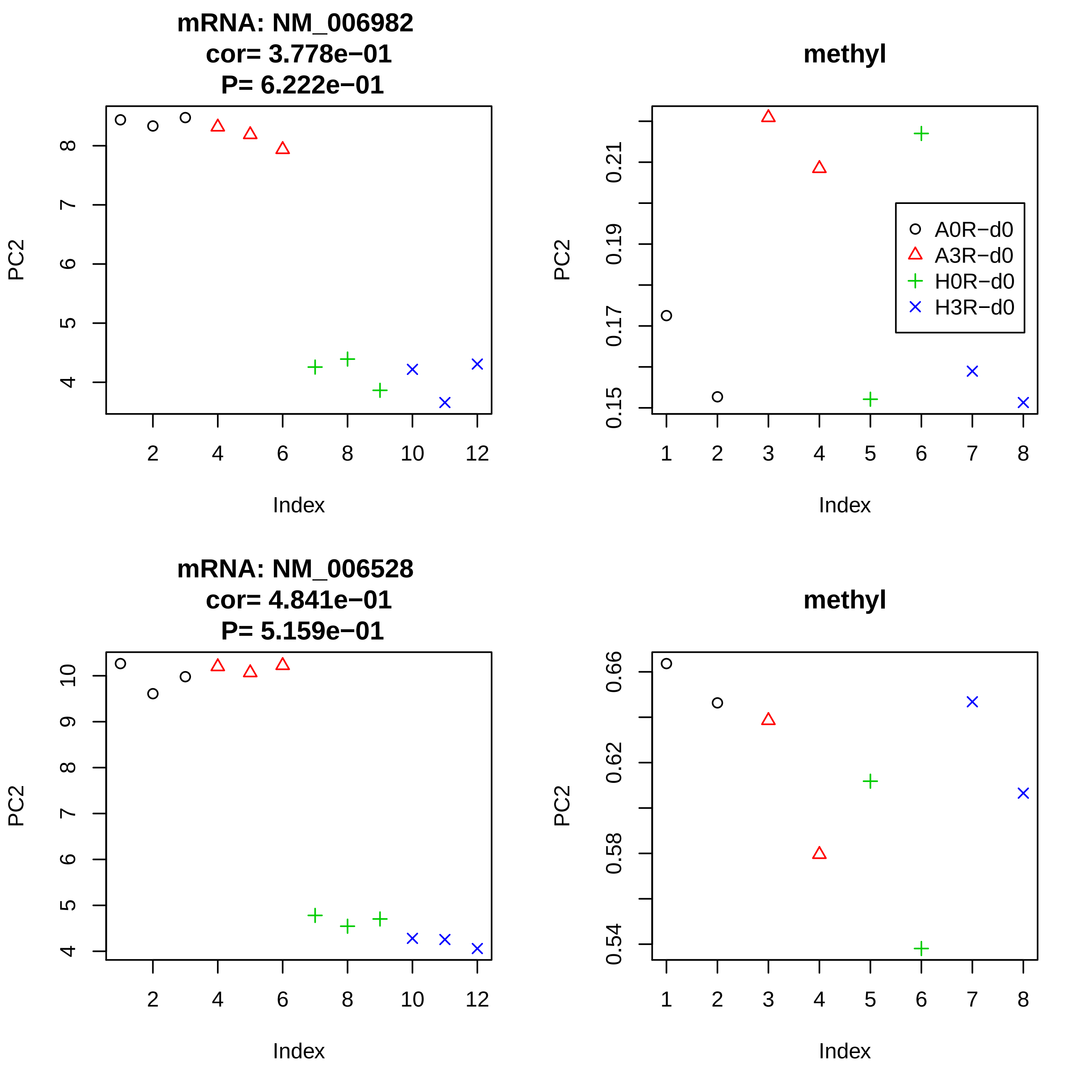

mRNA: NM_006982
cor= 3.778e−01
P= 6.222e−01
PC2
Index
methyl
PC2
Index
A0R−d0
A3R−d0
H0R−d0
H3R−d0
mRNA: NM_006528
cor= 4.841e−01
P= 5.159e−01
PC2
Index
methyl
PC2
Index
