# Supplementary material for: TINAGL1 and B3GALNT1 are potential therapy target genes to suppress metastasis in non-small cell lung cancer
Source: BMC Genomics. 2014 Dec 8;15(Suppl 9):S2. doi: 10.1186/1471-2164-15-S9-S2 (PMC4290609; doi:10.1186/1471-2164-15-S9-S2)
Supplement: Additional file 2 — Fig S2 Gene expression and promoter methylation associated with PC3. Gene expression and promoter methylation associated with PC3. Left column: gene expression, right column: promoter methylation. NM_002145 (HOXB2), NM_032040 (CCDC8), NM_153608 (ZNF114), NM_000793 (DIO2), NM_006762 (LAPTM5), NM_002922 (RGS1), NM_003781 (B3GALNT1). (Black open circles: A549 without metastasis, red triangles: A549 with metastasis, green crosses: HTB56 without metastasis, blue crosses: HTB56 with metastasis). Left column: gene expression, right column: promoter methylation. "cor" indicates Pearson correlation coefficients between gene expression and promoter methylation averaged within each of four categories and "P" is attributed to "cor". [file 1471-2164-15-S9-S2-S2.pdf]

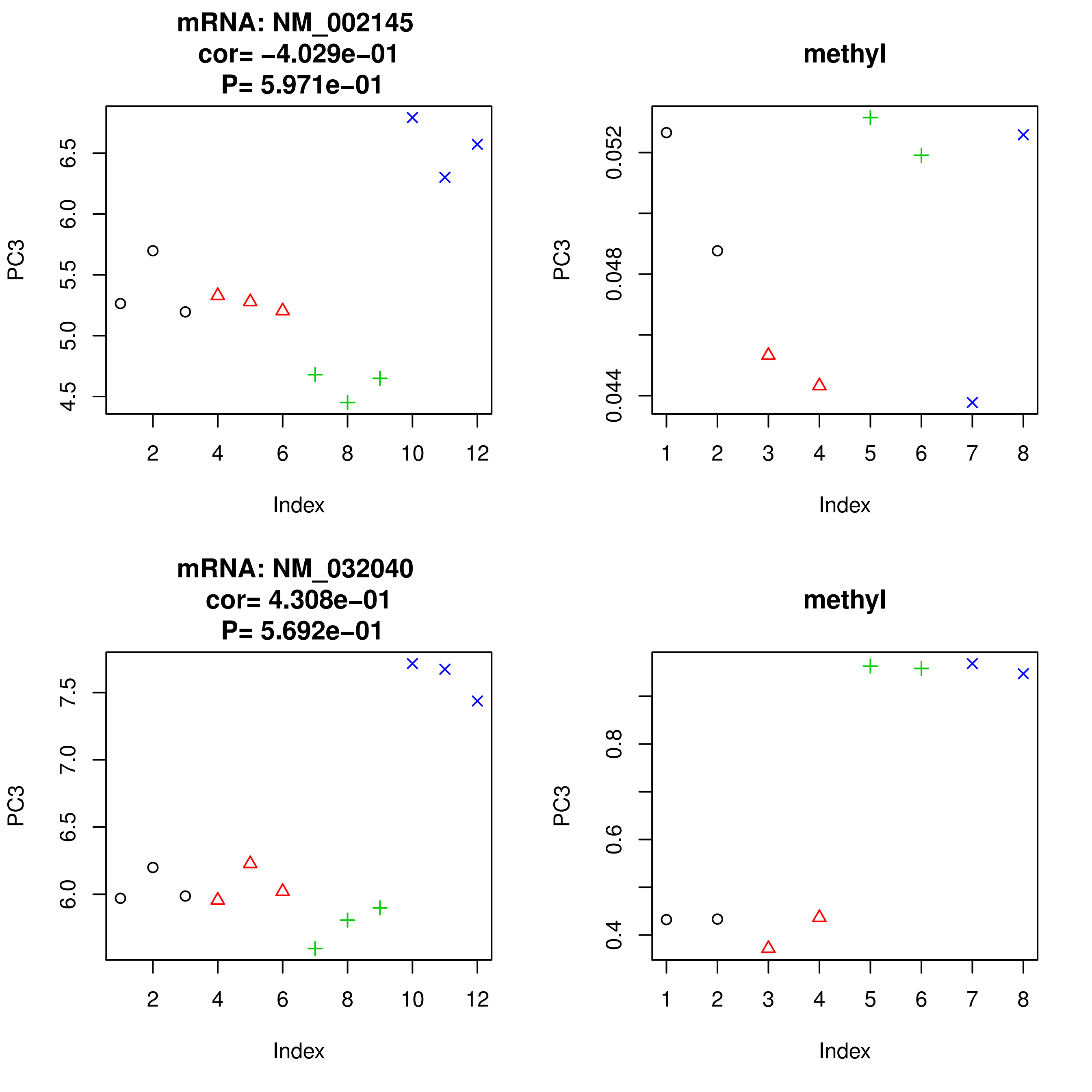
mRNA: NM_002145
cor= −4.029e−01
P= 5.971e−01
PC3
Index
methyl
PC3
Index
mRNA: NM_032040
cor= 4.308e−01
P= 5.692e−01
PC3
Index
methyl
PC3
Index

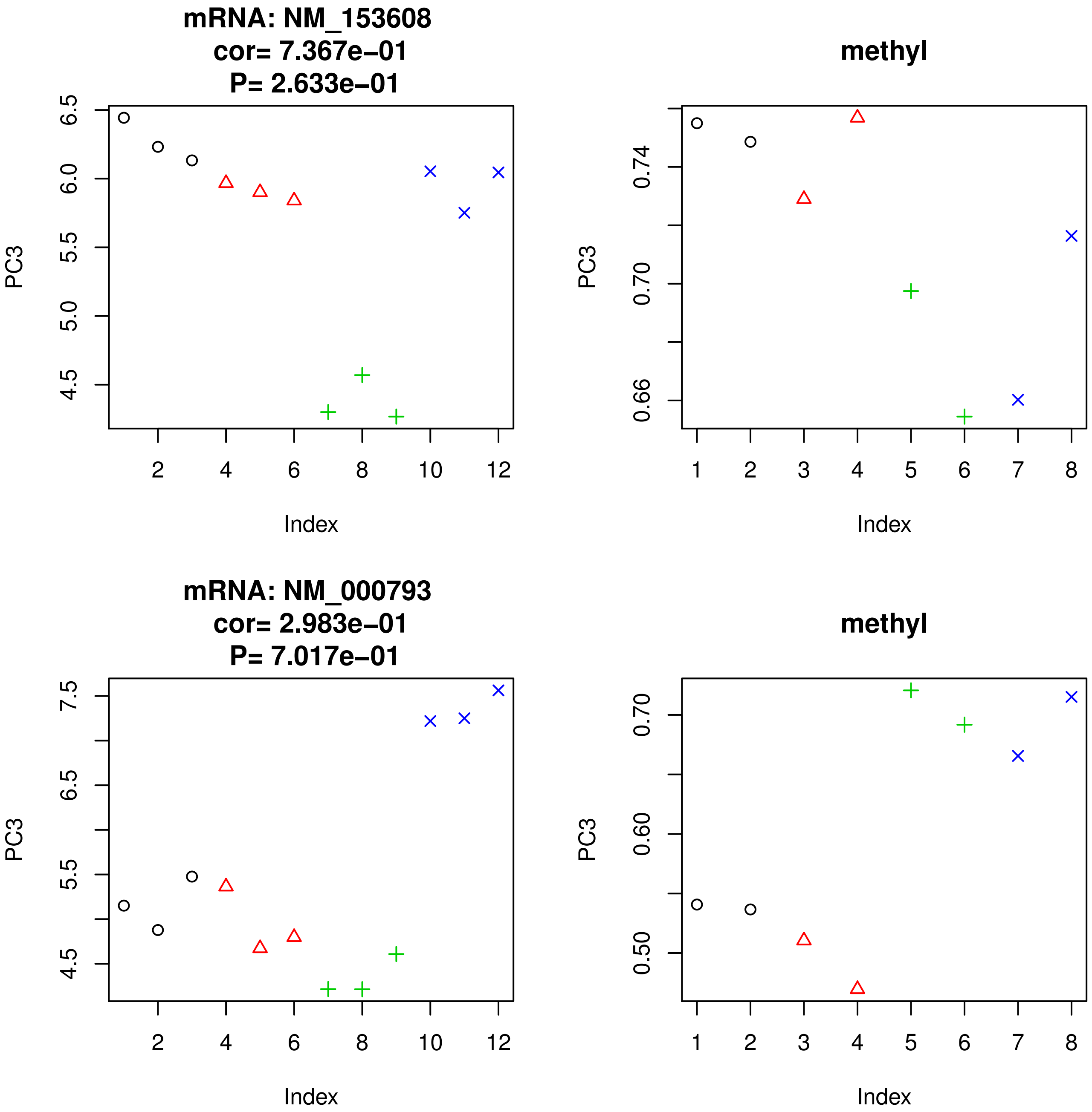
mRNA: NM_153608
cor= 7.367e−01
P= 2.633e−01
PC3
6.5
6.0
5.5
5.0
4.5
2
4
6
8
10
12
Index
methyl
PC3
0.74
0.70
0.66
1
2
3
4
5
6
7
8
Index
mRNA: NM_000793
cor= 2.983e−01
P= 7.017e−01
PC3
7.5
6.5
5.5
4.5
2
4
6
8
10
12
Index
methyl
PC3
0.70
0.60
0.50
1
2
3
4
5
6
7
8
Index

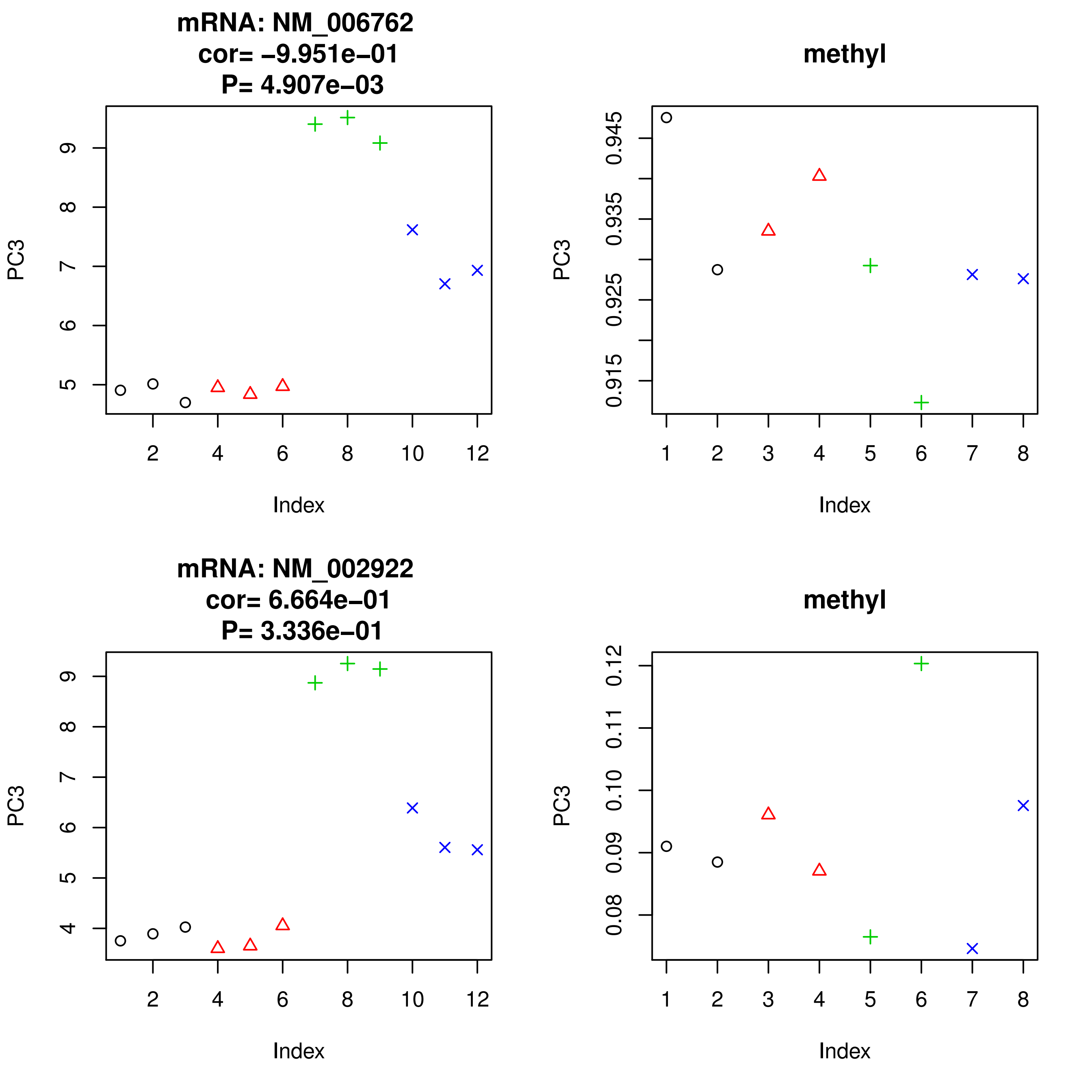

mRNA: NM_006762
cor= −9.951e−01
P= 4.907e−03
methyl
PC3
Index
mRNA: NM_002922
cor= 6.664e−01
P= 3.336e−01
methyl
PC3
Index

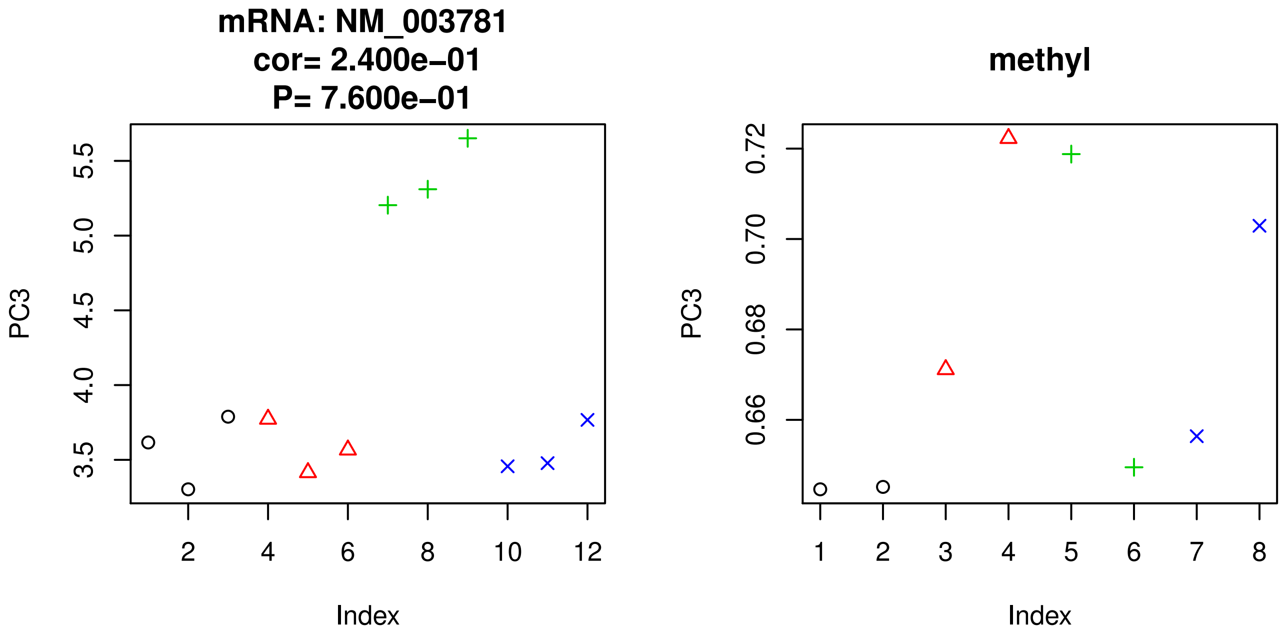

mRNA: NM_003781
cor= 2.400e−01
P= 7.600e−01
PC3
5.5
5.0
4.5
4.0
3.5
2
4
6
8
10
12
Index
methyl
PC3
0.72
0.70
0.68
0.66
1
2
3
4
5
6
7
8
Index
