# Supplementary figures and images for: TINAGL1 and B3GALNT1 are potential therapy target genes to suppress metastasis in non-small cell lung cancer
Source: BMC Genomics. 2014 Dec 8;15(Suppl 9):S2. doi: 10.1186/1471-2164-15-S9-S2 (PMC4290609; doi:10.1186/1471-2164-15-S9-S2)

mRNA: NM\_022164

cor= 7.503e-01

P= 2.497e-01

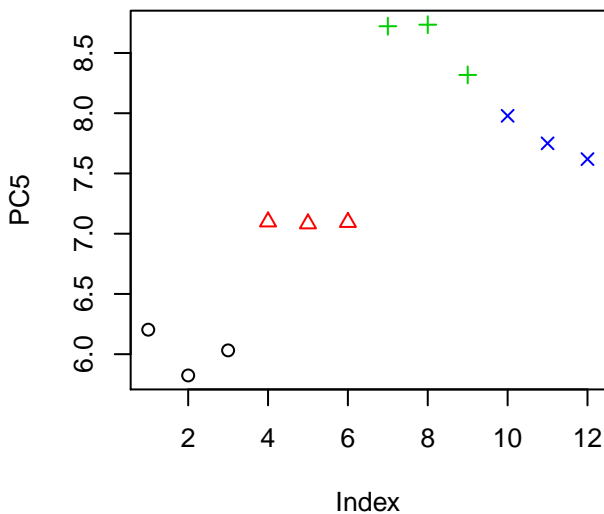

methyl

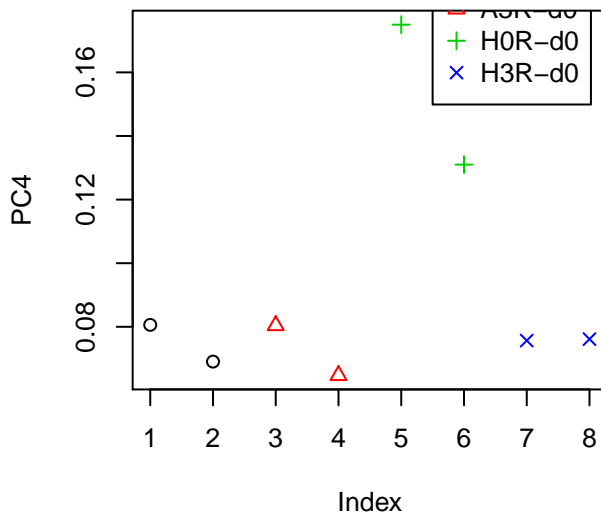

mRNA: NM\_020182

cor= -8.313e-01

P= 1.687e-01

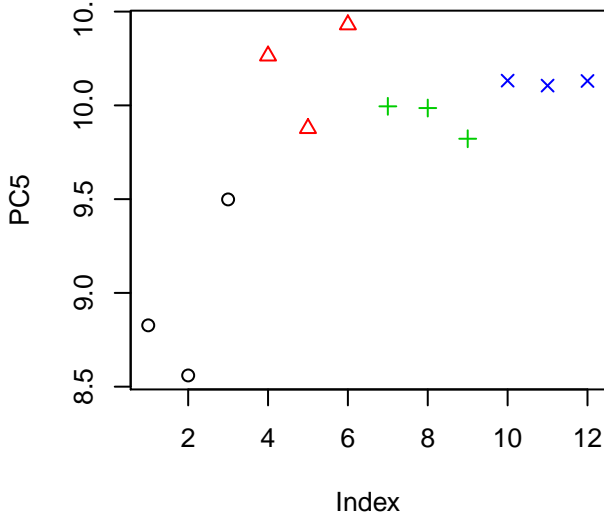

methyl

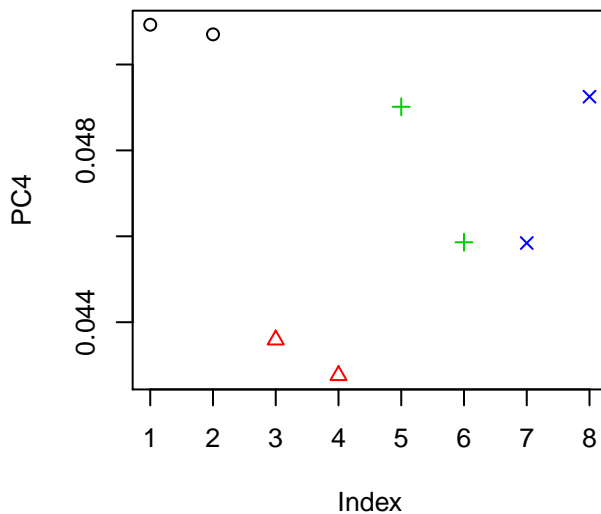

**mRNA: NM\_002996**

**cor=  $-5.678e-01$**

**P=  $4.322e-01$**

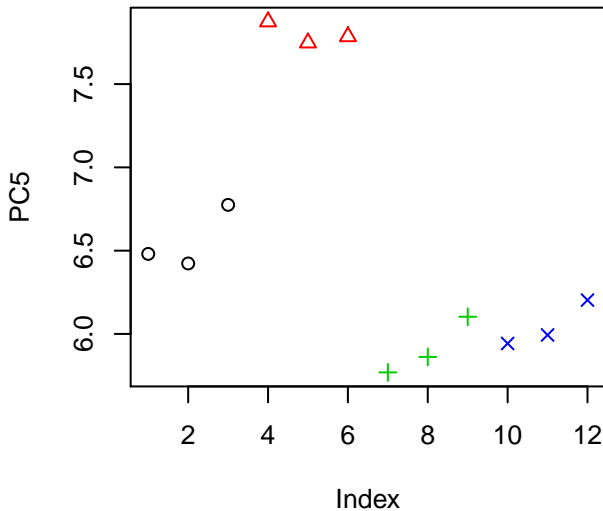

**methyl**

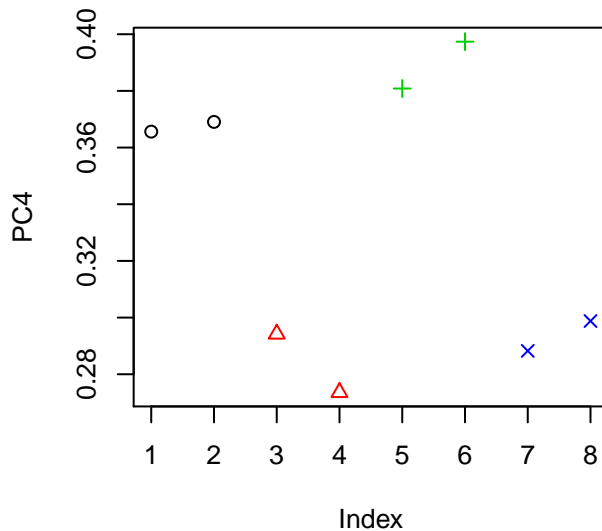

**mRNA: NM\_000201**

**cor=  $2.224e-01$**

**P=  $7.776e-01$**

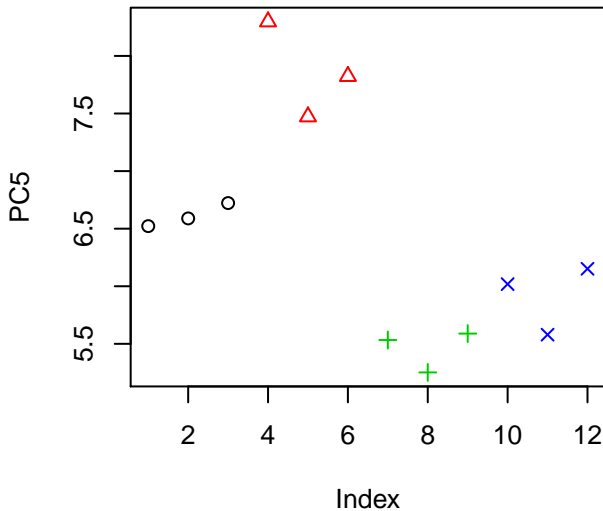

**methyl**

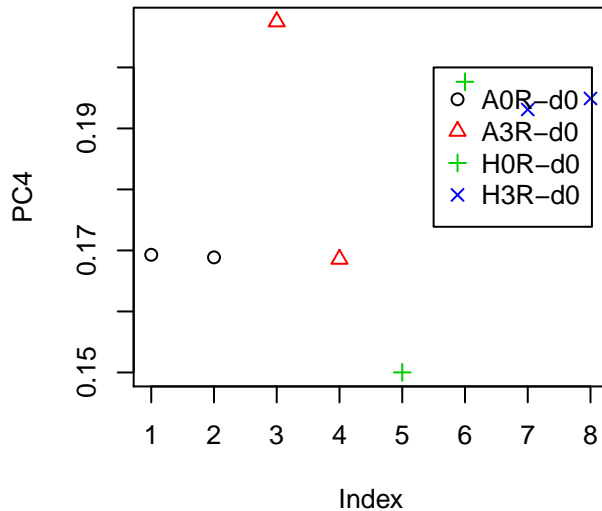

Supplement: Additional file 3 — Fig. S3 Gene expression and promoter methylation associated with PC4 (promoter methylation) and PC5 (gene expression). Gene expression and promoter methylation associated with PC4 (promoter methylation) and PC5 (gene expression). Left column: gene expression, right column: promoter methylation. NM_022164 (TINAGL1), NM_020182 (PMEPA1), NM_002996 (CX3CL1), NM_000201 (ICAM1). Contributions of samples (black open circles: A549 without metastasis, red triangles: A549 with metastasis, green crosses: HTB56 without metastasis, blue crosses: HTB56 with metastasis) to PCs. Left column: gene expression, right column: promoter methylation. "cor" indicates Pearson correlation coefficients between gene expression and promoter methylation averaged within each of four categories and "P" is attributed to "cor". [file 1471-2164-15-S9-S2-S3.pdf]

**methy1 NM\_002145**

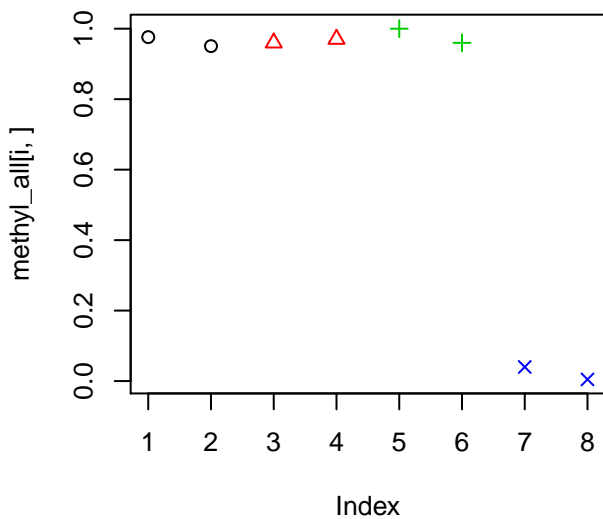

**methy1 NM\_032040**

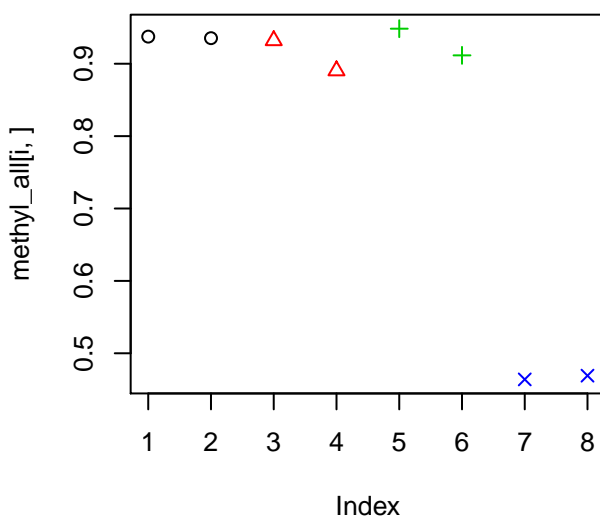

**methy1 NM\_153608**

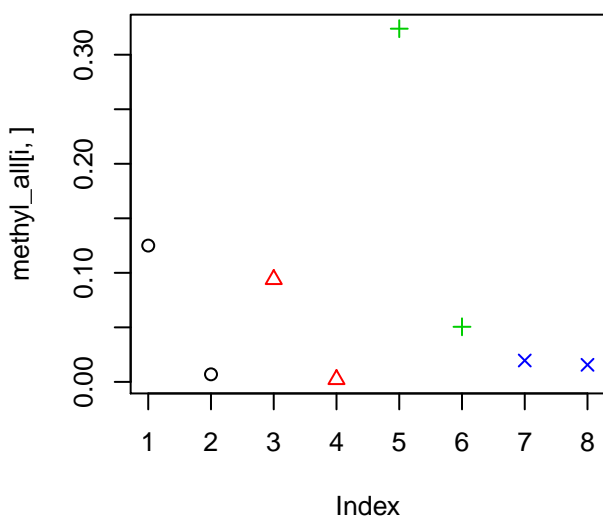

**methy1 NM\_006762**

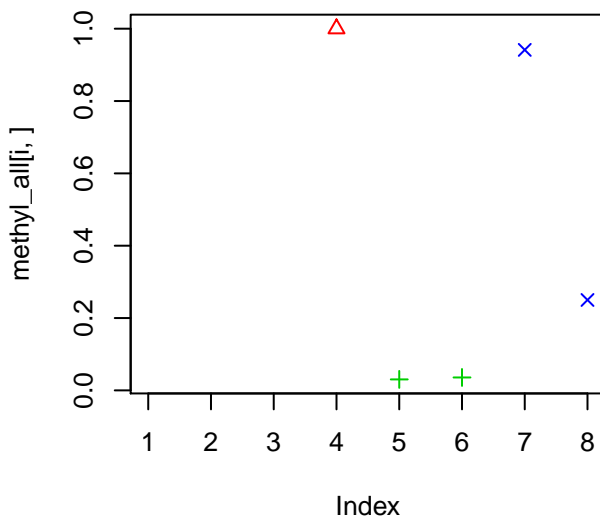

**methyl NM\_003781**

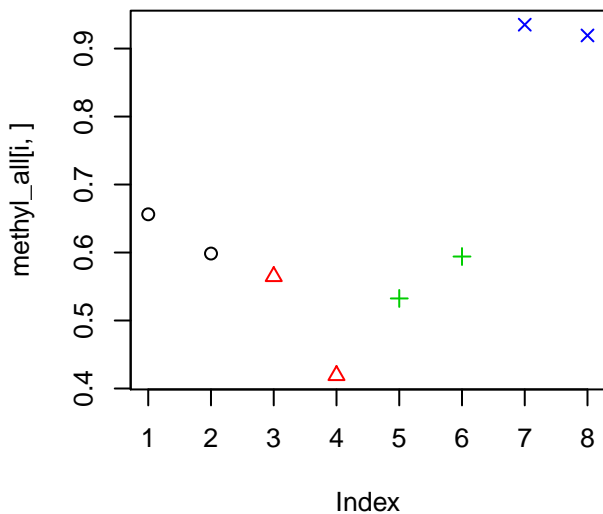

**methyl NM\_022164**

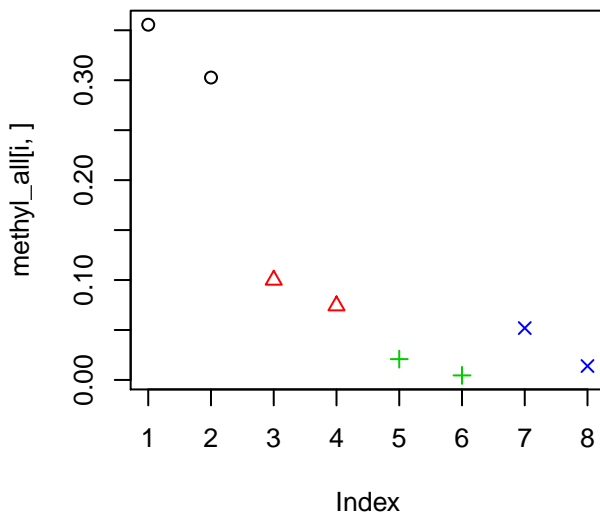

**methyl NM\_020182**

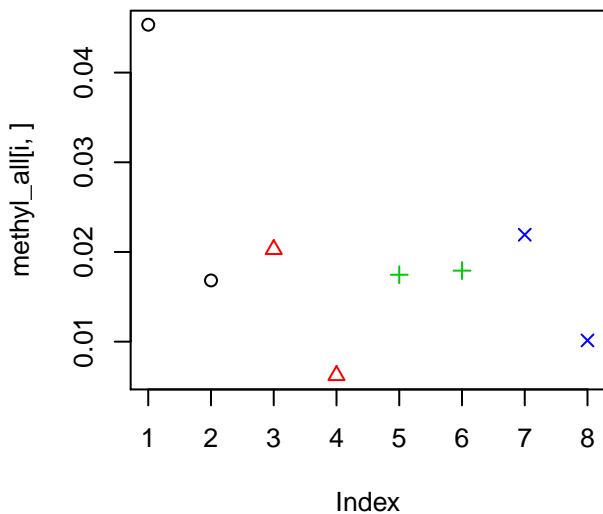

**methyl NM\_002996**

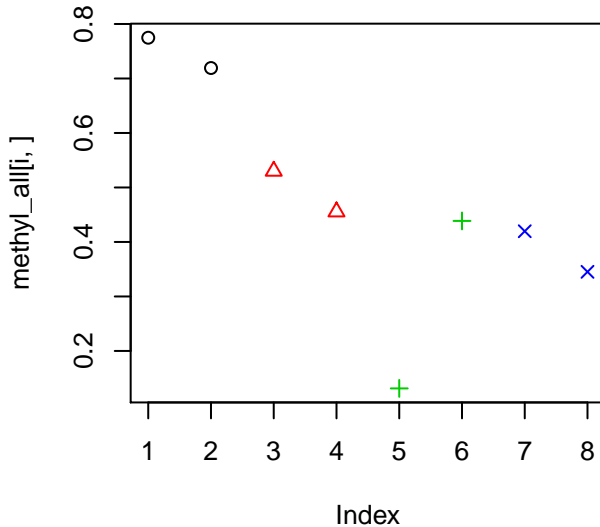

**methyl NM\_000201**

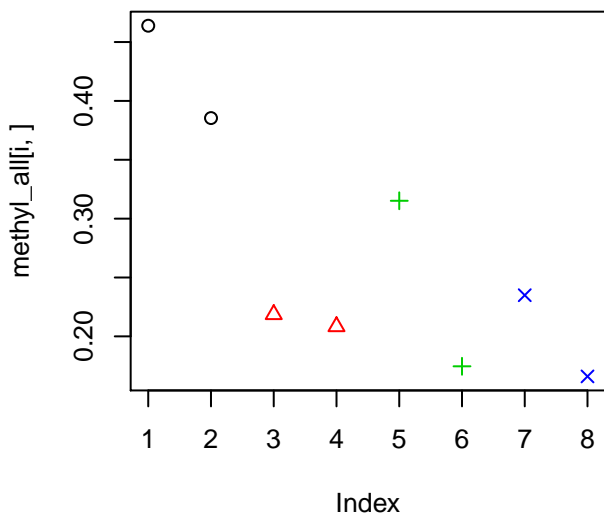

Supplement: Additional file 6 — Fig. S15 Promoter methylation profile of selected genes measured by sequencing technology. Promoter methylation measured by sequencing, NM_002145 (HOXB2), NM_032040 (CCDC8), NM153608 (ZNF114), NM_006762 (LAPTM5), NM_003781 (B3GALNT1), NM_022164 (TINAGL1), NM_020182 (PMEPA1), NM_002996 (CX3CL1), and NM_000201 (ICAM1). [file 1471-2164-15-S9-S2-S6.pdf]
