# Supplementary material for: TINAGL1 and B3GALNT1 are potential therapy target genes to suppress metastasis in non-small cell lung cancer
Source: BMC Genomics. 2014 Dec 8;15(Suppl 9):S2. doi: 10.1186/1471-2164-15-S9-S2 (PMC4290609; doi:10.1186/1471-2164-15-S9-S2)
Supplement: Additional file 4 — Supplementary figures. Supplementary figures from Fig. S4 to Fig. S14. [file 1471-2164-15-S9-S2-S4.pdf]

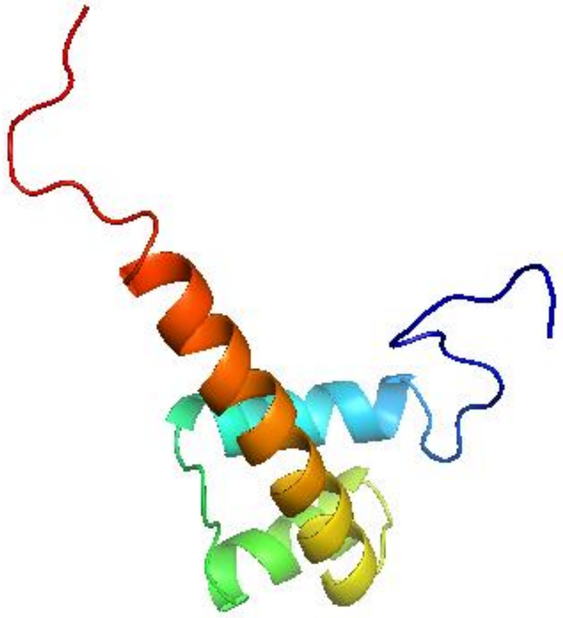

Figure S4 - Tertiary structure of Homeobox domain of *HOXB2* predicted by FAMS using PDB ID: 2CUE_A as a reference.

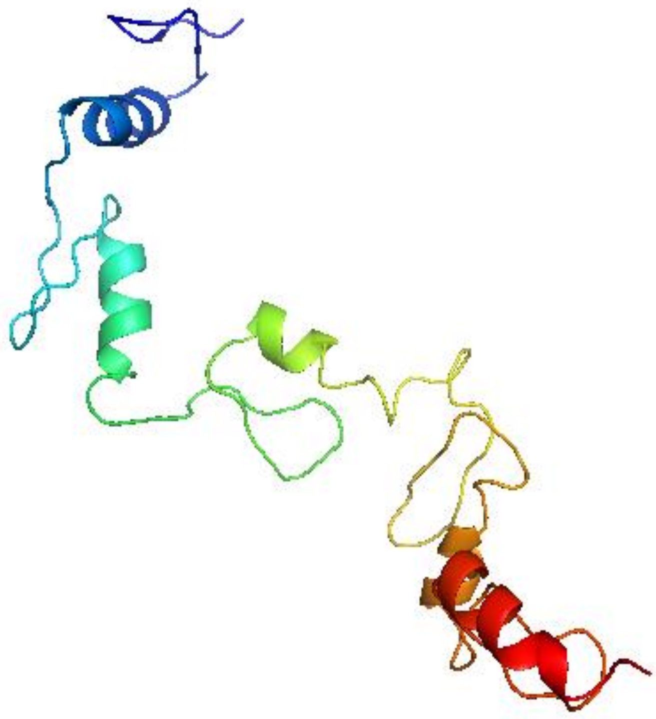

Figure S5 - Tertiary structure of Zinc finger domains of *ZNF114* predicted by FAMS using PDB ID: 1TF6_A as a reference.

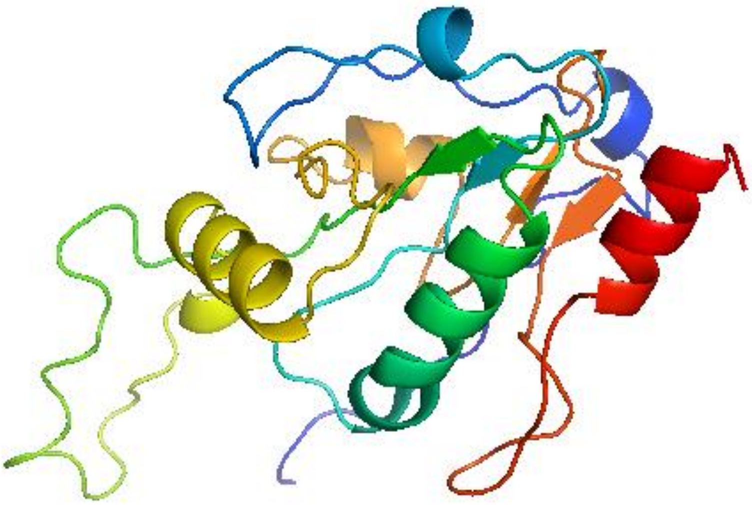

Figure S6 - Tertiary structure of *DIO2* predicted by FAMS using PDB ID: 2YWI_A as a reference.

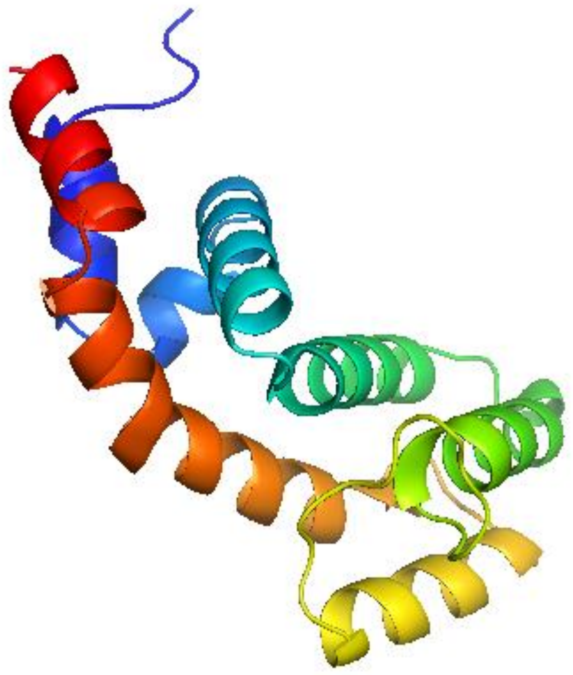

Figure S7 - Tertiary structure of *RGS1* in Protein Data Bank.

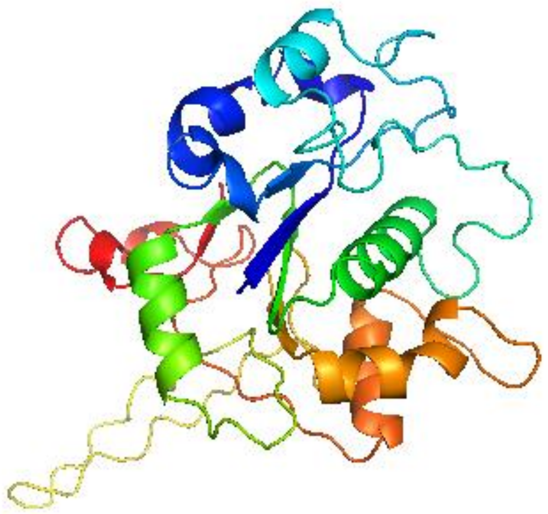

Figure S8 - Tertiary structure of *B3GALNT1* predicted by FAMS using PDB ID: 2J0A_A as a reference.

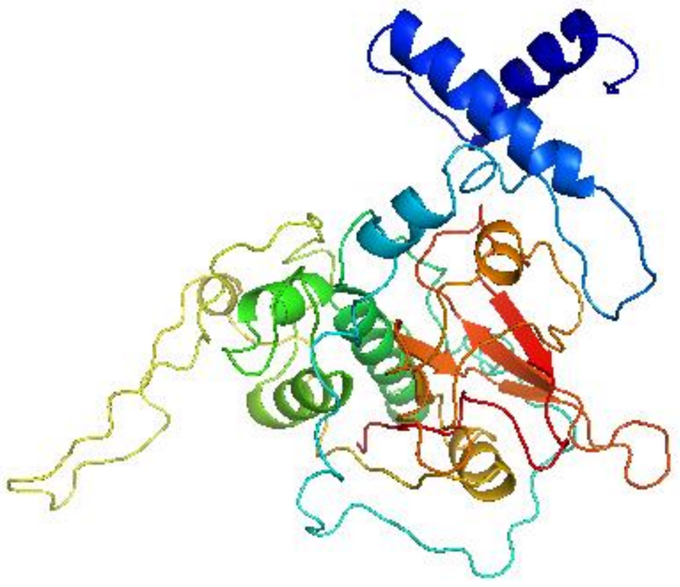

Figure S9 - Tertiary structure of *TIANGL1* predicted by FAMS using PDB ID: 3TNX_A as a reference.

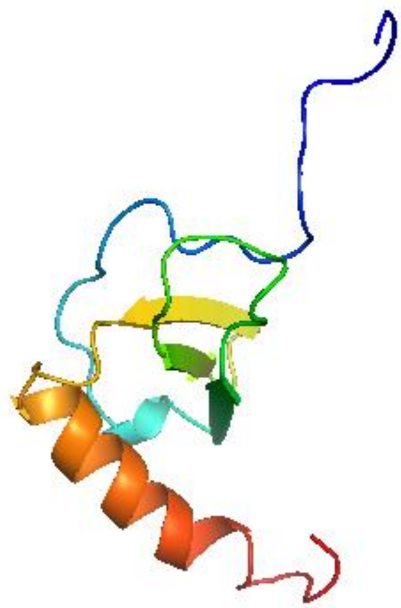

Figure S10 - Tertiary structure of IL-8 domain in *CX3CL1* predicted by FAMS using PDB ID: 1B2T_A as a reference.

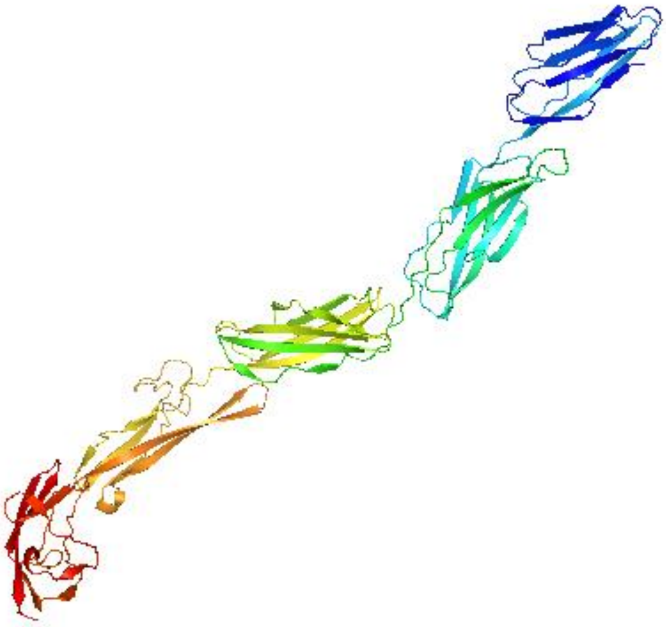

Figure S11 - Tertiary structure of *ICAM1* in Protein Data Bank.

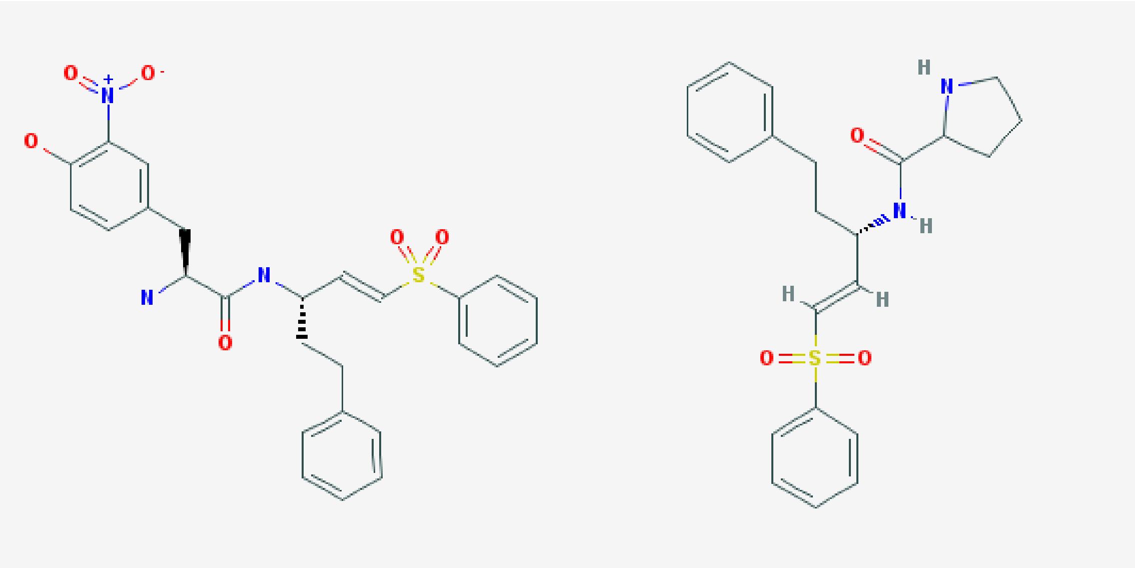

Figure S12 - Drug candidate compounds that bind to *TINAGL1*.

Left: CHEMBL1242746, right: CHEMBL1242747.

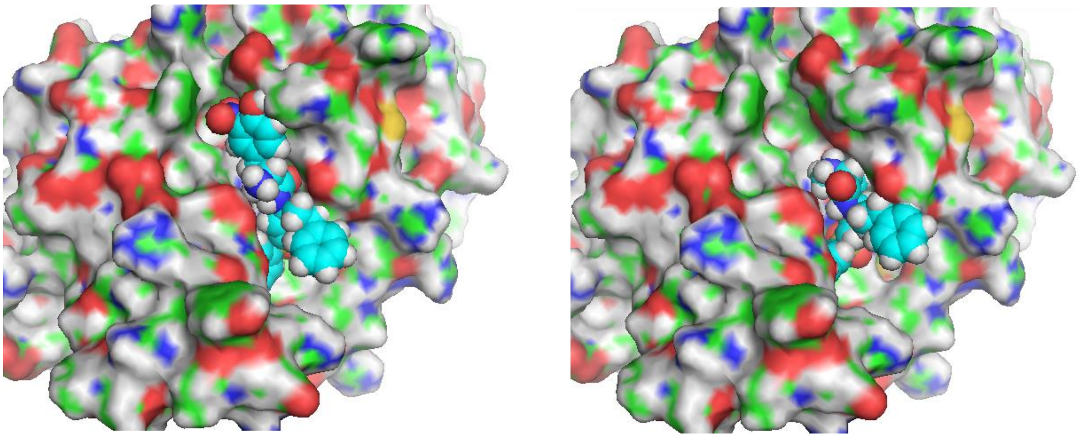

Figure S13 - Ligand candidates binding to cathepsin domain in *TINAGL1* inferred by chooseLD.

Left: CHEMBL1242746, right: CHEMBL1242747. Red: oxygen, blue: nitrogen, white: hydrogen, orange: sulphur, and green (cyan): carbon.

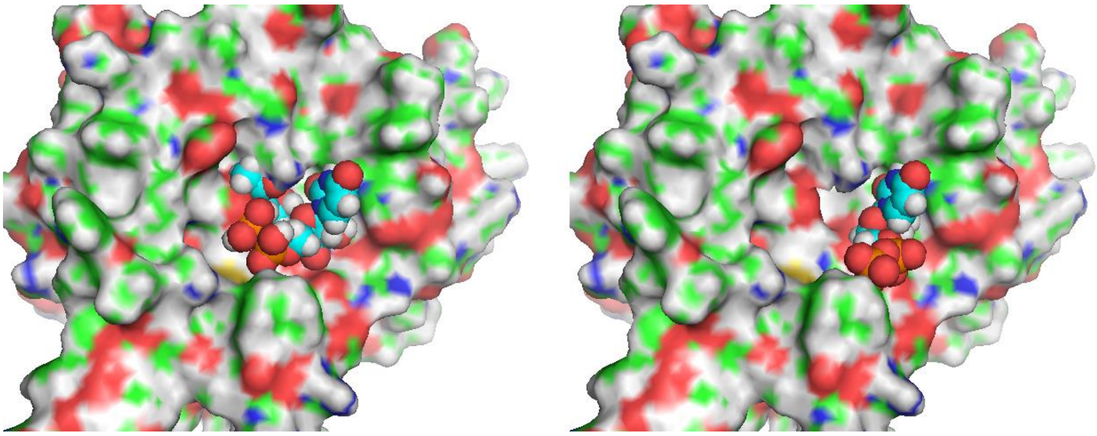

Figure S14 - Ligands that bind to *B3GALNT1* inferred by chooseLD.

Left: UDP-N-acetylglucosamine, Right: UDP. Red: oxygen, blue: nitrogen, white: hydrogen, orange: sulphur, and green (cyan): carbon.
