# Supplementary material for: TINAGL1 and B3GALNT1 are potential therapy target genes to suppress metastasis in non-small cell lung cancer
Source: BMC Genomics. 2014 Dec 8;15(Suppl 9):S2. doi: 10.1186/1471-2164-15-S9-S2 (PMC4290609; doi:10.1186/1471-2164-15-S9-S2)

1GMY_hld, Cyscore = -1.8086

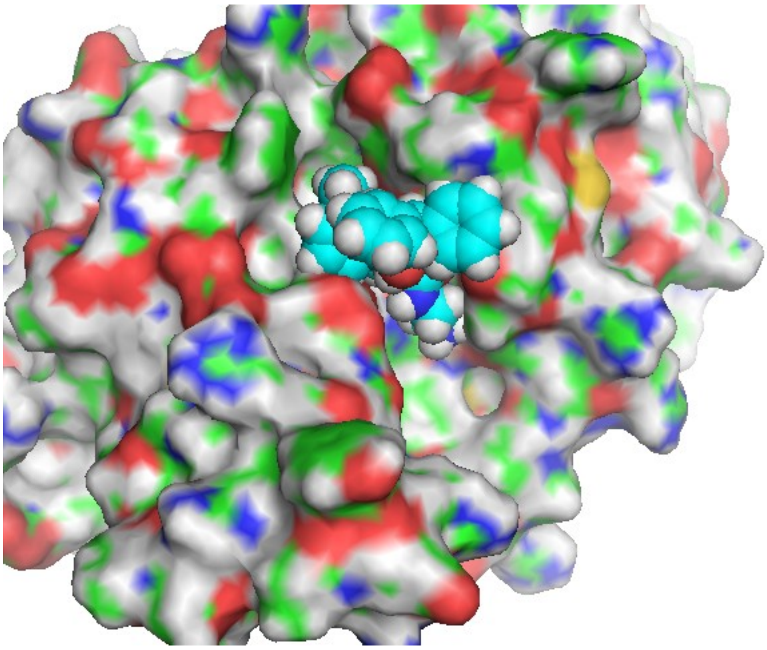

1ITO_E6C, Cyscore = -0.4461

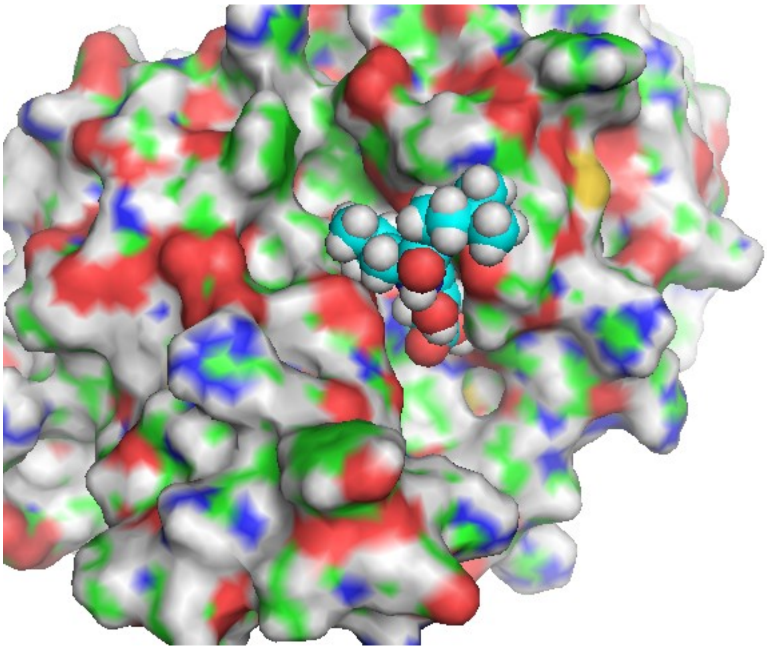

1QDQ_074, Cyscore=-2.8243

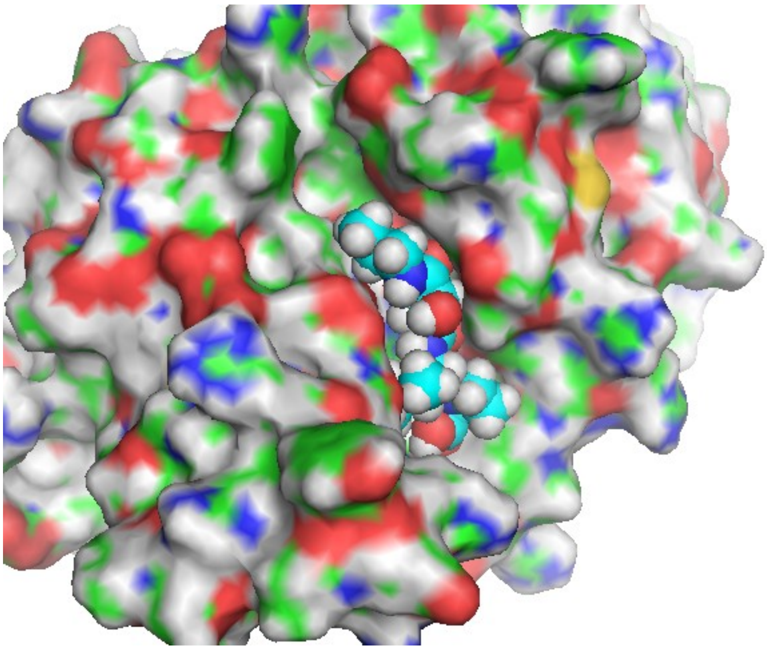

2DC6_73V, Cyscore= -1.7081

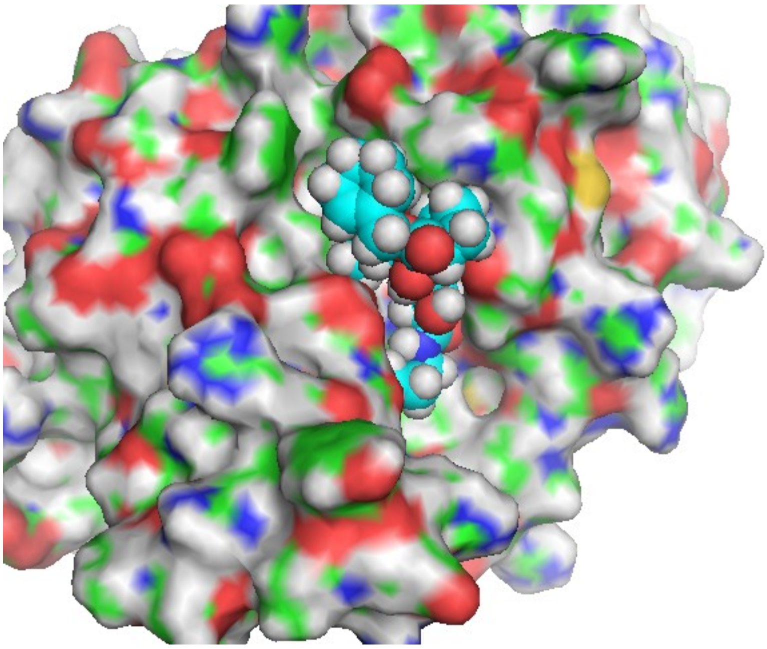

2DC7_042, Cyscore= -1.514

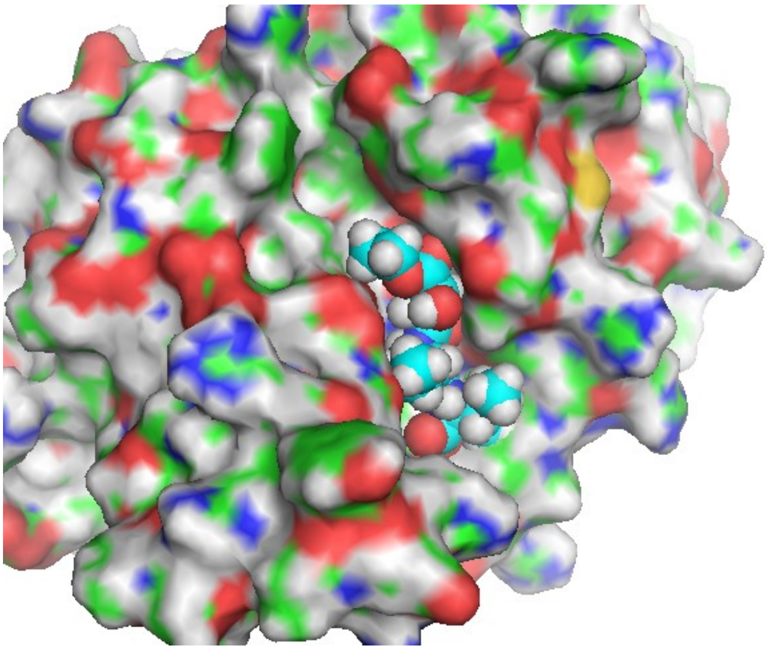

2DC8_59A, Cyscore=-1.717

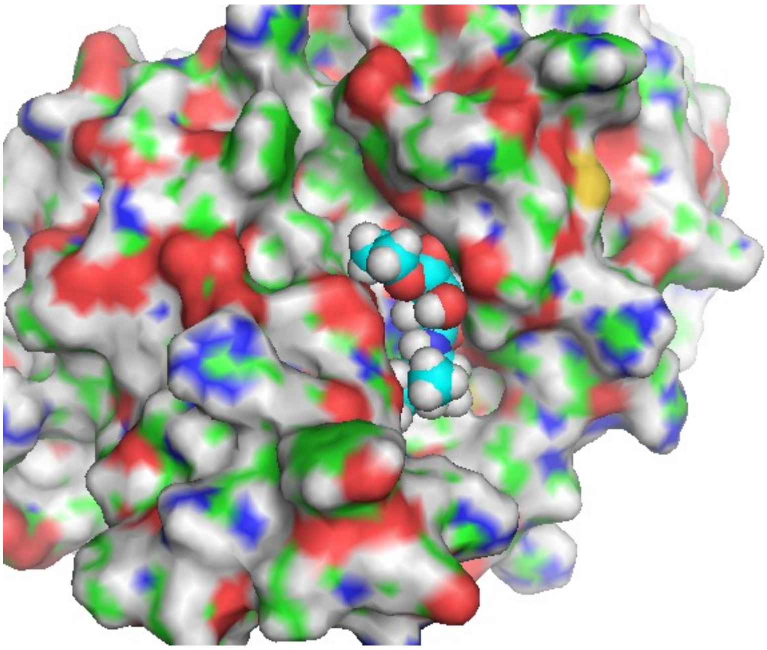

2DC9_74M, Cyscore=-2.1026

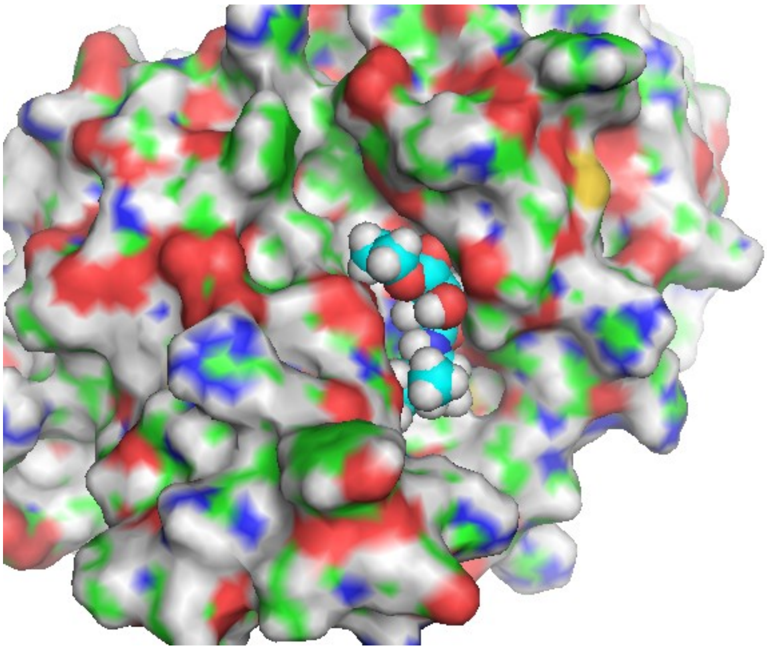

2DCA_75V, Cyscore=-2.1103

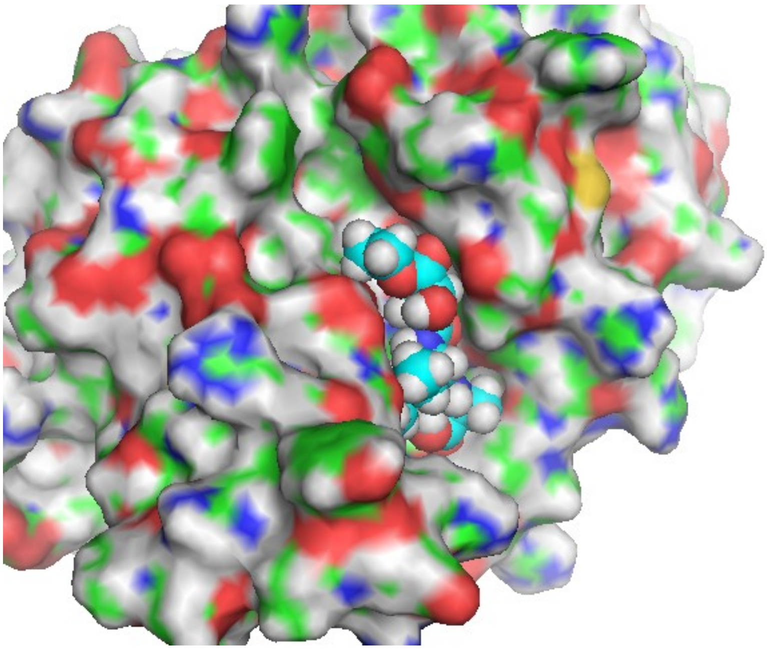

2DCB_76V, Cyscore=-2.7419

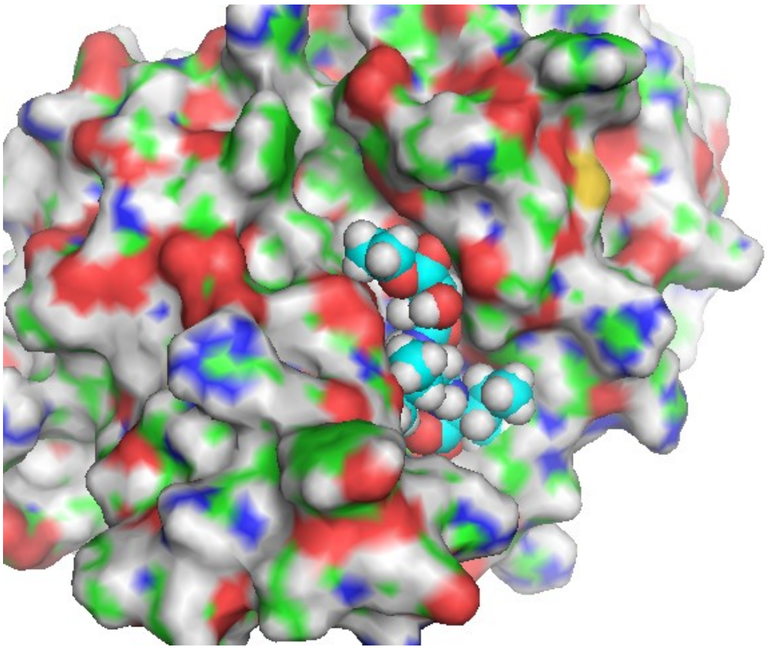

2DCC_77B, Cyscore= -2.0373

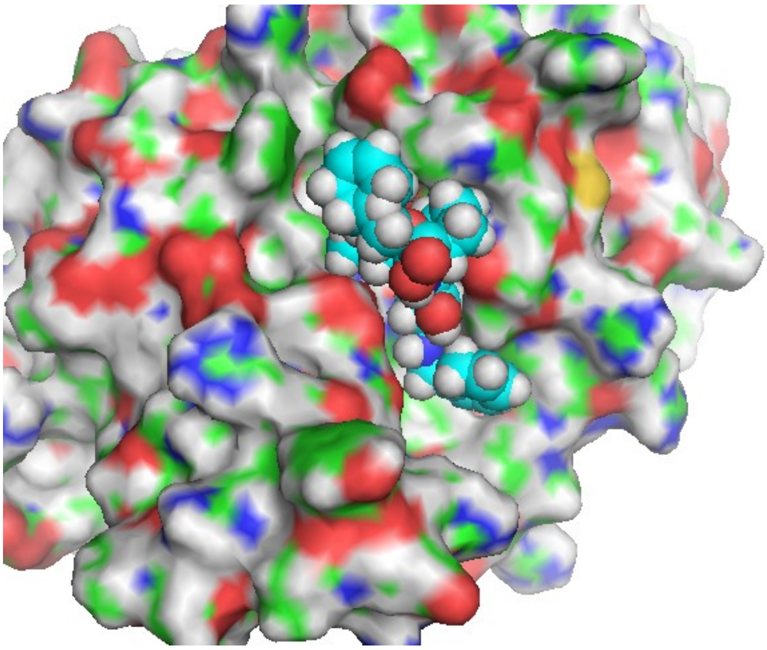

2DCD_78A, Cyscore=-2.7627

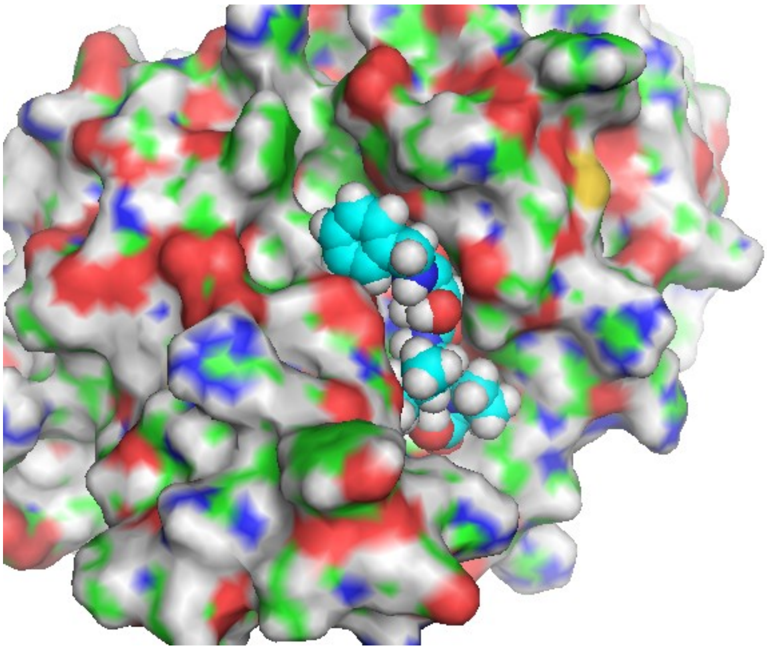

3AI8_HNQ, Cyscore= -0.0704

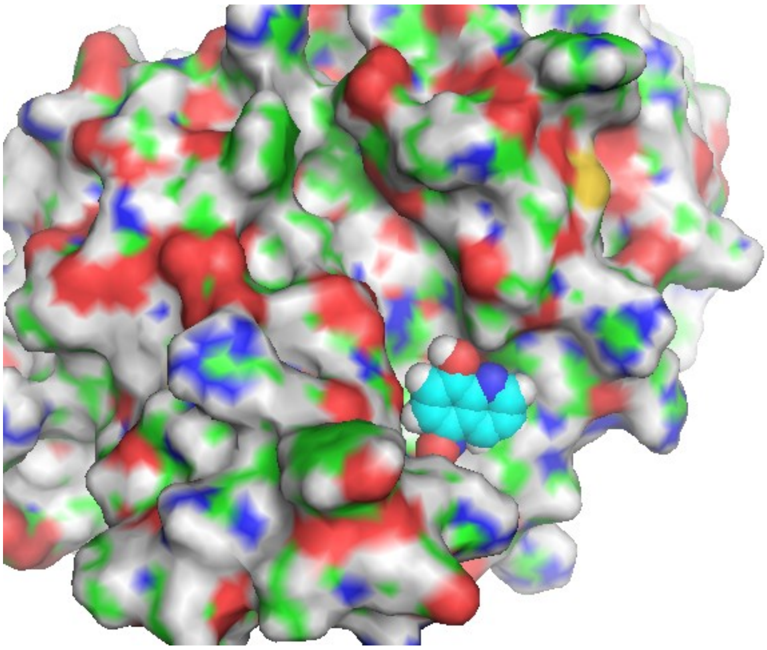

3PDF_LXV, Cyscore=-1.4434

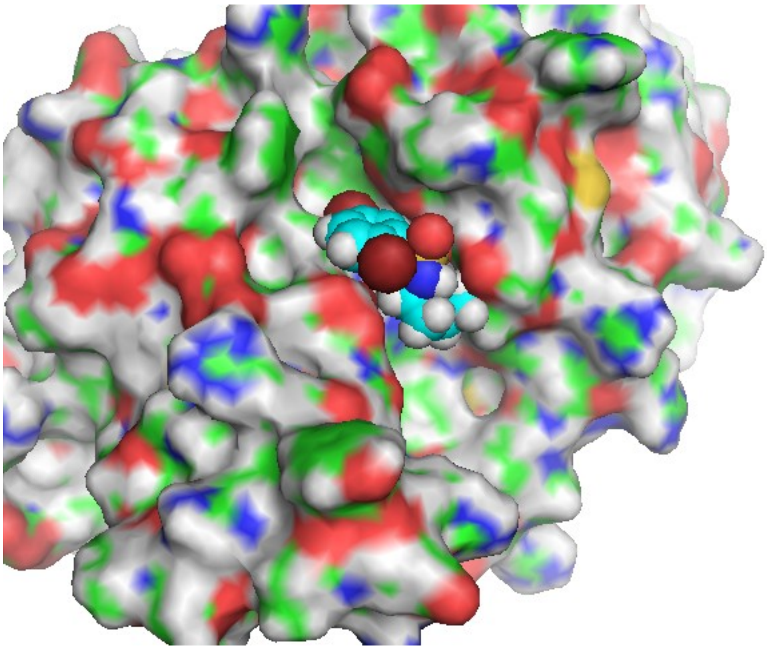

3S3Q_C1P, Cyscore= -2.4019

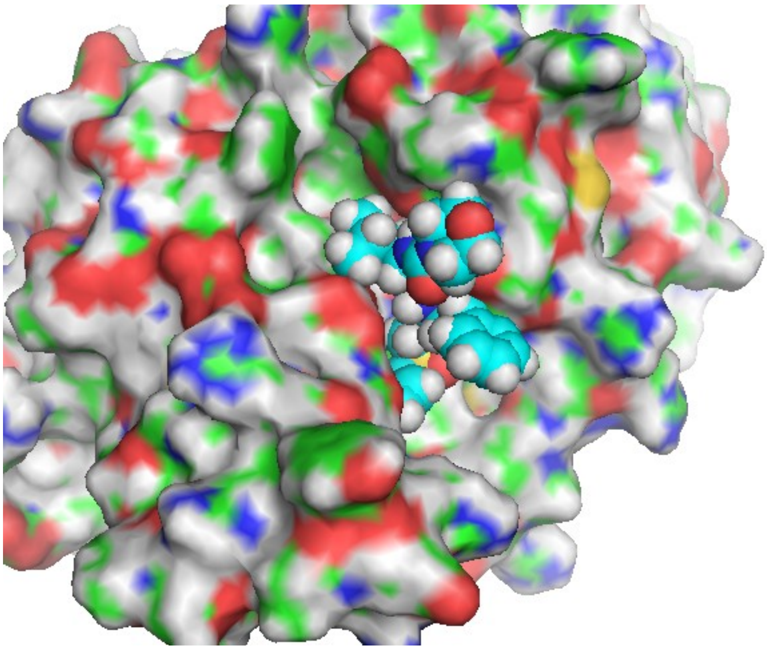

3S3R_0IW, Cyscore=-1.5028

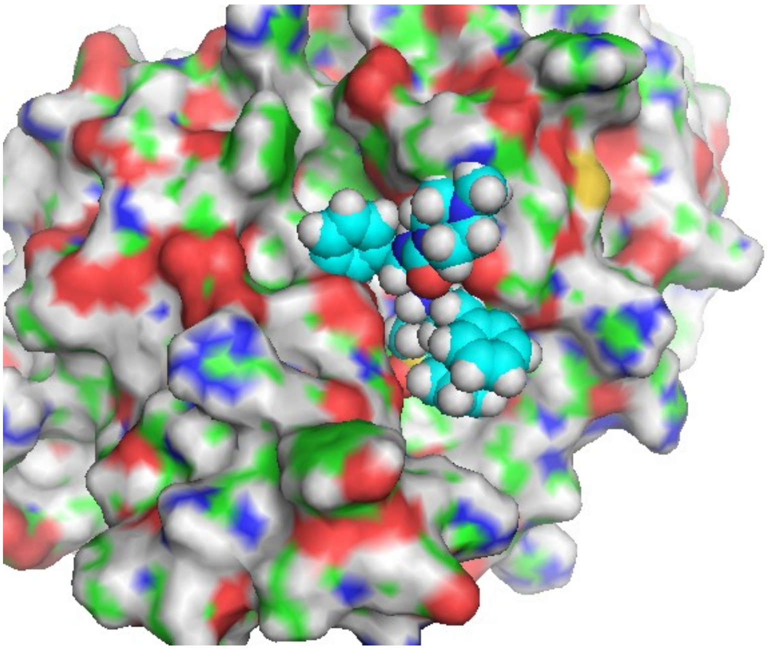

CHEMBL1242746, Cyscore= 1.8177

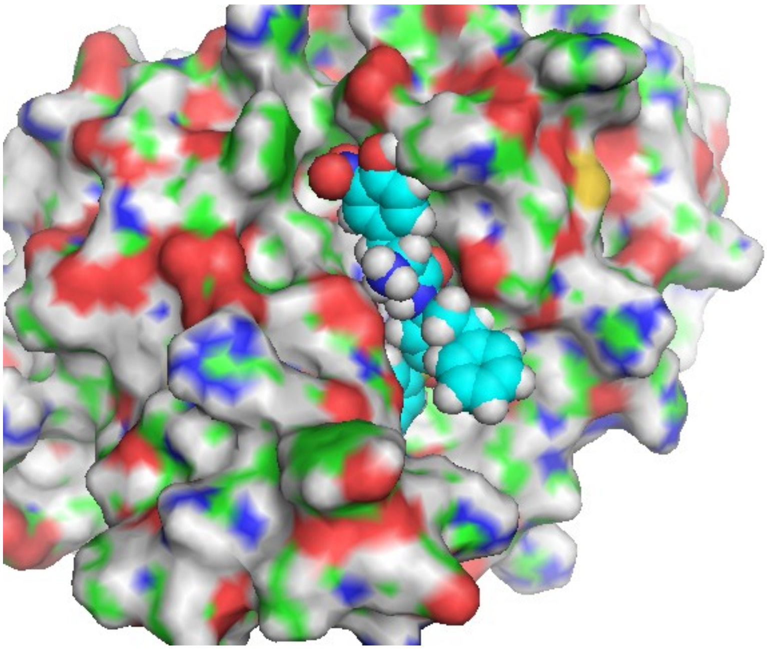

CHEMBL1242747, Cyscore=0.206

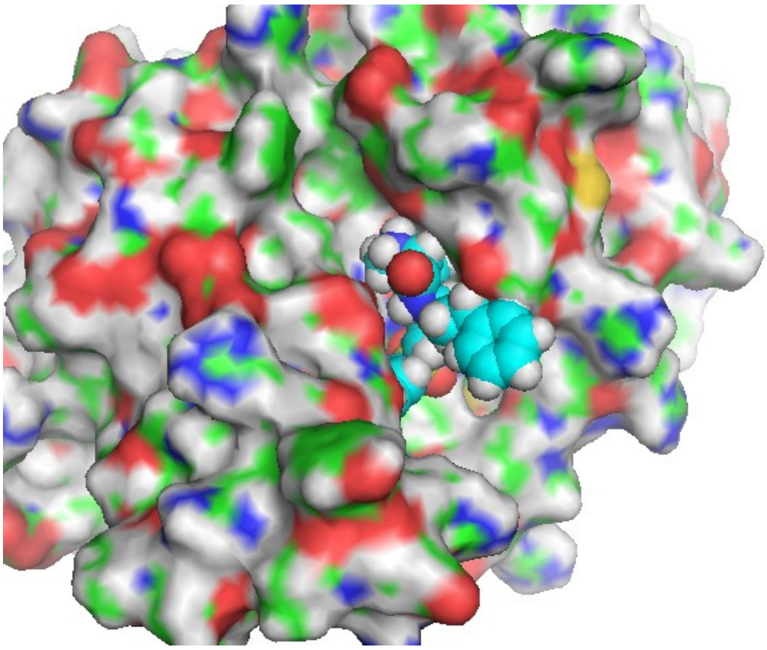

Supplement: Additional file 5 — Ligand binding configuration to TINAGL1. Full list of ligands that bind to TINAGL1. [file 1471-2164-15-S9-S2-S5.pdf]
